# Optimizing uPAR-targeting radiopeptides for improved tissue distribution: progress towards radionuclide therapy

**DOI:** 10.1007/s00259-025-07602-7

**Published:** 2025-10-27

**Authors:** Christian Vaccarin, Darja Beyer, Jerome V. Schmid, Bastian Klein, Jathursa Jegathasan, Shreshtha Behera, Xavier Deupi, Roger Schibli, Cristina Müller

**Affiliations:** 1 Center for Radiopharmaceutical Sciences, PSI Center for Life Sciences, Villigen-PSI, 5232 Switzerland; 2 Condensed Matter Theory Group, PSI Center for Scientific Computing, Theory and Data, Villigen-PSI, 5232 Switzerland; 3 Laboratory of Biomolecular Research, PSI Center for Life Sciences, Villigen-PSI, 5232 Switzerland; 4 Swiss Institute of Bioinformatics (SIB), Lausanne, 1015 Switzerland; 5 Department of Chemistry and Applied Biosciences, ETH Zurich, Zurich, 8093 Switzerland

**Keywords:** Urokinase-type plasminogen activator receptor, uPAR, Lutetium-177, SPECT imaging, Albumin binder, DOTA-AE105, *p*-tolyl entity

## Abstract

**Purpose:**

The aim of this study was to develop radiopeptides for targeting the urokinase-type plasminogen activator receptor (uPAR) modified with an albumin-binding moiety to improve their tissue distribution profiles.

**Methods:**

uPAR-11, uPAR-12, uPAR-14, uPAR-15, uPAR-17 and uPAR-18 were synthesized based on the AE105 nonapeptide which was modified with the *p*-tolyl-based albumin binder and variable linker entities and chelators. The ^177^Lu-labeled peptides were evaluated in vitro with regard to their stability, albumin-binding properties and uPAR-binding affinity using HEK-uPAR cells. Biodistribution and SPECT/CT imaging studies were performed with HEK-uPAR xenografted nude mice. The acquired data were compared to those obtained with [^177^Lu]Lu-DOTA-AE105.

**Results:**

The radiopeptides showed 11‒155-fold higher albumin-binding affinity in human blood plasma than [^177^Lu]Lu-DOTA-AE105. The uPAR-binding affinity reached K_D_ values of 31‒42 nM, similar to the K_D_ value of 20 ± 1 nM determined for [^177^Lu]Lu-DOTA-AE105. Accumulation in the HEK-uPAR xenograft was 6.0–16% IA/g at 4 h p.i., which was 7‒18-fold higher than that of [^177^Lu]Lu-DOTA-AE105. PEG spacers next to the albumin binder as exemplified in [^177^Lu]Lu-uPAR-11, [^177^Lu]Lu-uPAR-15 and [^177^Lu]Lu-uPAR-18 led to increased xenograft accumulation while their replacement with an alkane spacer in [^177^Lu]Lu-uPAR-12 led to unfavorably high blood retention. A diaminopropionic acid to connect the different entities did not improve the tissue distribution profile of [^177^Lu]Lu-uPAR-14 as compared to other peptides which were designed with a lysine residue. The use of a DOTAGA chelator instead of a DOTA resulted in unfavorable kidney retention of [^177^Lu]Lu-uPAR-17.

**Conclusion:**

In view of a clinical translation, [^177^Lu]Lu-uPAR-11 emerged as the most favorable candidate. Future studies will, thus, focus on the therapeutic potential of [^177^Lu]Lu-uPAR-11 in tumor-bearing mice.

**Graphical abstract:**

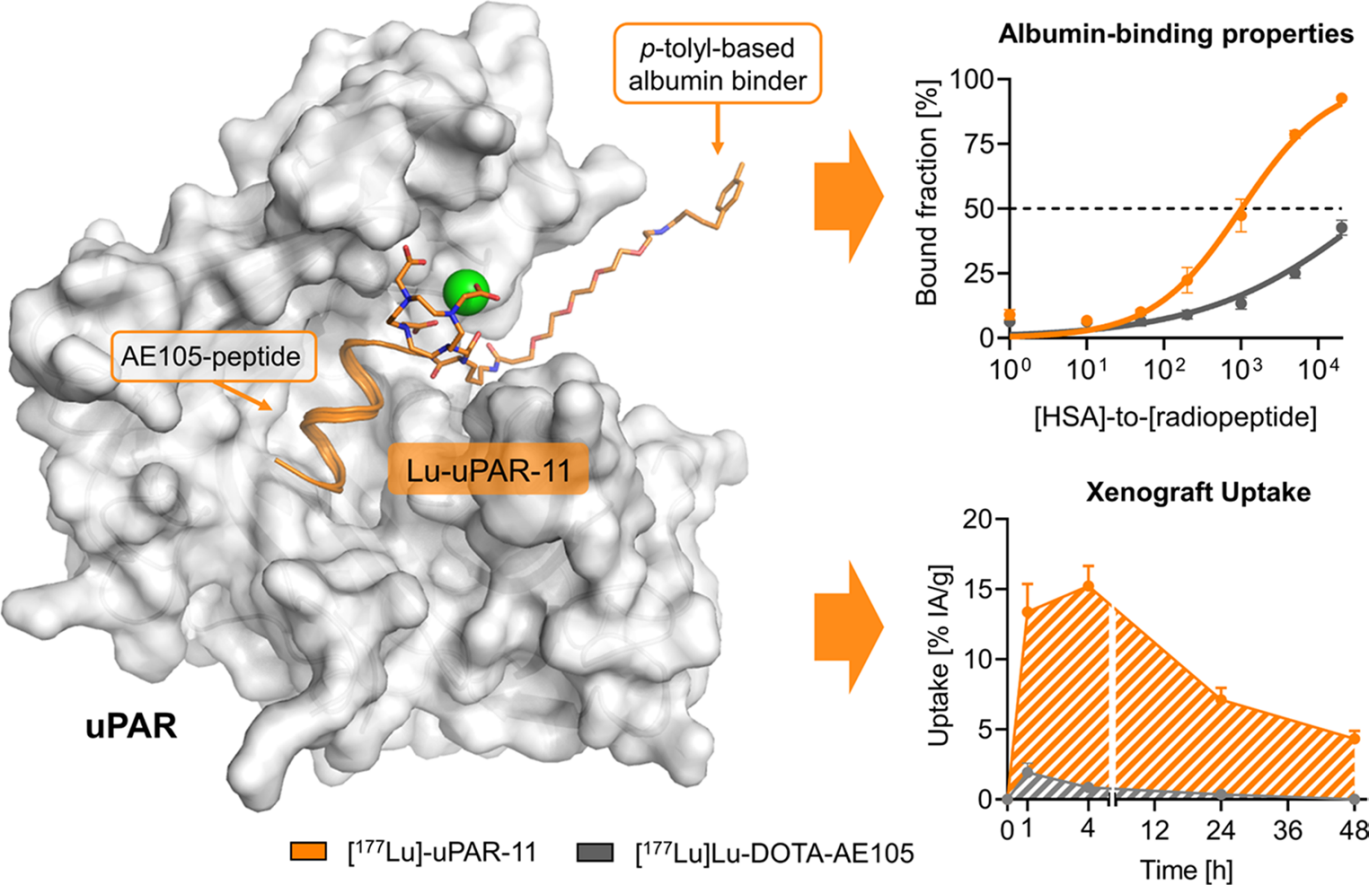

**Supplementary Information:**

The online version contains supplementary material available at 10.1007/s00259-025-07602-7.

## Introduction

The human urokinase-type plasminogen activator receptor (uPAR) is a cell-associated protein anchored to the cell membrane via a glycosylphosphatidylinositol entity. uPAR regulates pericellular proteolysis by recruiting urokinase-type plasminogen activator (uPA) to the cell surface, where it binds to uPAR and converts plasminogen to plasmin, thereby mediating the degradation of the extracellular matrix components [1]. As a result, uPAR plays a pivotal role in biological processes that require tissue remodeling, such as embryonal development and wound healing [2]. Although uPAR expression is limited in healthy tissue, it is upregulated in a wide range of cancers, where it is found on tumor cells and tumor-associated stromal cells [3–8]. uPAR expression in cancer positively correlates with tumor progression, invasion and metastatic potential and is typically associated with aggressive tumor phenotypes and poor clinical outcomes [9, 10]. Therefore, uPAR may be an attractive target for the development of tumor-targeting imaging agents and radiotherapeutics.

The nonapeptide AE105 was previously identified as a uPAR-binding peptide useful for tumor-targeting [11]. AE105 originated from a phage-display selection of a peptide library [12], followed by a medicinal chemistry optimization process aimed at improving its affinity for uPAR. AE105 acts as an antagonist by binding to the lipophilic uPA-binding pocket of uPAR with a binding affinity of 0.36 nM and, hence, blocks the uPA-uPAR association [13]. AE105 has been functionalized with several macrocyclic chelators to enable the coordination of radionuclides and the use of the resultant radiopeptides for nuclear imaging of uPAR-expressing cancer. Among these, DOTA-AE105 and NOTA-AE105 were combined with gallium-68 or copper-64 for preclinical investigations using positron emission tomography (PET) [14, 15], followed by translation of [^64^Cu]Cu-DOTA-AE105 and [^68^Ga]Ga-DOTA-AE105 to initial clinical trials [14, 16–18]. The rapid blood clearance of these radiopeptides was found to be advantageous for diagnostic imaging in cancer patients, as it enabled high tumor-to-background contrast already short after radiopeptide administration. Nevertheless, the rapid blood clearance together with the reported low in vivo stability of these radiopeptides are unfavorable features in view of a therapeutic application and may have been the reason for the limited treatment efficacy of [^177^Lu]Lu-DOTA-AE105 in tumor-bearing mice [15, 19]. Recently, we have developed a series of novel radiopeptides (uPAR-01, uPAR-02, uPAR-03, uPAR-04 and uPAR-05) based on AE105 and modified with the *p*-iodophenyl entity as a high-affinity albumin binder using various linker entities [20]. Such modifications enhanced the blood circulation time of the resultant uPAR-targeting radiopeptides in mice and, consequently, led to an increased xenograft accumulation and retention [21]. [^177^Lu]Lu-uPAR-02 modified with a polyethylene glycol-4 (PEG_4_) spacer to connect the *p*-iodophenyl entity with the DOTA-AE105 backbone emerged as the most promising candidate with more than 10-fold higher accumulation in uPAR-expressing xenografts compared to that of [^177^Lu]Lu-DOTA-AE105 at 4 h after injection. In normal tissue, such as the liver and kidneys, the accumulation of [^177^Lu]Lu-uPAR-02 was only minimally increased compared to the accumulation of [^177^Lu]Lu-DOTA-AE105. The considerable blood retention may, however, comprise a risk of damage to bone marrow cells following administration of [^177^Lu]Lu-uPAR-02 at therapeutic activities. Similar situations were previously observed for prostate-specific membrane antigen (PSMA)-targeting radioligands modified with a *p*-iodophenyl entity which led to extensive blood retention in mice [22, 23]. The replacement of this albumin binder with the *p*-tolyl entity resulted in a considerably improved tissue distribution profile of the resultant PSMA radioligand ([^177^Lu]Lu-PSMA-ALB-56) [23], which could be ascribed to the 10-fold lower albumin-binding affinity of the *p*-tolyl entity as compared to that of the *p*-iodophenyl entity [20]. Similar to the example of the design of PSMA radioligands, we aimed to develop a next generation of uPAR-targeting radiopeptides incorporating the *p*-tolyl group as an albumin-binding entity.

**Fig. 1:**
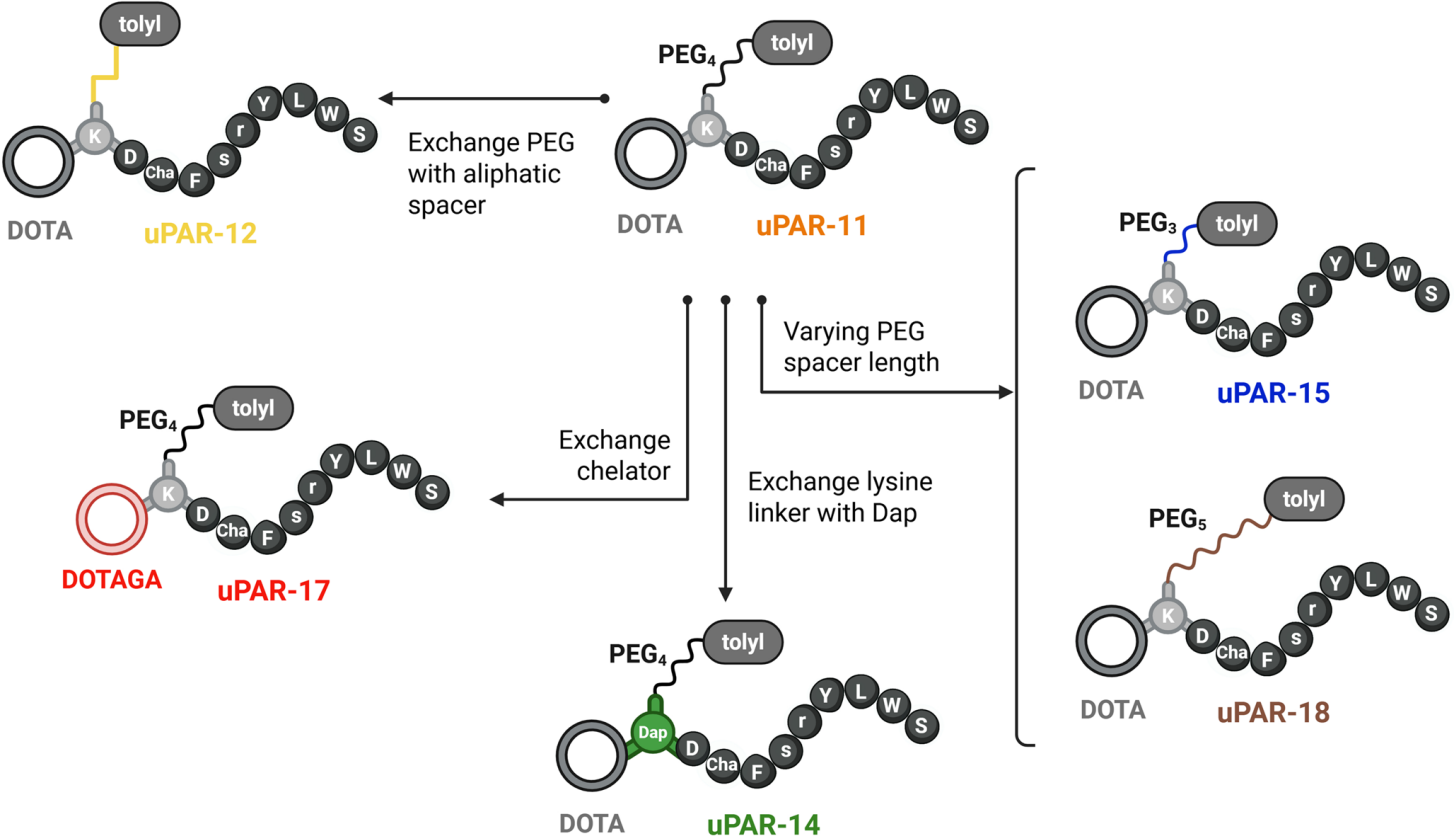
Schematic representation of the uPAR-targeting peptides with a *p*-toly-entity as an albumin binder. The peptide sequence is presented using the one-letter amino acids code (Cha, l-cyclohexylalanine; Dap, l-diaminopropionic acid). Created in BioRender. Beyer, D. (2025)

Six new peptides were designed based on AE105 as the uPAR-targeting agent and a *p*-tolyl entity conjugated via various linker entities (Fig. 1). uPAR-11 was designed with an analogous chemical structure to that of uPAR-02, with a PEG_4_ spacer introduced between the lysine residue and the *p*-tolyl entity. Further modifications regarding the length of the PEG spacer (PEG_3_ and PEG_5_) resulted in uPAR-15 and uPAR-18, respectively. In uPAR-12, these PEG spacers were replaced by an aliphatic entity next to the albumin binder. Finally, the exchange of the interconnecting lysine residue with a diaminopropionic acid residue led to the design of uPAR-14, while the replacement of the DOTA chelator with DOTAGA resulted in uPAR-17. These peptides were labeled with lutetium-177, and the resultant radiopeptides were preclinically evaluated to identify the candidate with the most favorable pharmacokinetic properties in view of a therapeutic application.

## Methods

### Synthesis of the uPAR-targeting peptides

The uPAR-targeting peptides were synthesized according to a previously reported procedure (Supplementary Material, Fig. S1, Scheme S1) [21]. In brief, the resin-immobilized and side-chain-protected AE105 scaffold was synthesized using standard solid-phase peptide synthesis coupling methodologies. The *Nα*-terminus of the peptide chain was modified with a lysine or Dap residue. The selective deprotection of the *N*_ɛ_ group of the lysine residue or the *N*_β_ group of the Dap, respectively, enabled the conjugation of a PEG (PEG_3_, PEG_4_ or PEG_5_) or aliphatic (8-aminooctanoic acid) spacer followed by the attachment of 4-(*p*-tolyl)butyric acid. The *N*_α_ group of the lysine or Dap residue was deprotected and conjugated with a 2-(4,7,10-tris(carboxymethyl)−1,4,7,10-tetrazacyclododec-1-yl)acetic acid (DOTA) or 2-(4,7,10-tris(carboxymethyl)−1,4,7,10-tetraazacyclododecan-1-yl)pentanedioic acid (DOTAGA) chelator. After cleavage from the resin and global deprotection, the crude peptides were purified using semipreparative high performance liquid chromatography (HPLC) (Supplementary Material, Table S1). The chemical purity of the final compounds was determined by analytical HPLC, while their chemical identity was confirmed by high-resolution mass spectrometry (HRMS).

### Preparation and stability of the uPAR-targeting radiopeptides

The radiolabeling of the peptides was carried out at molar activities of 5‒50 MBq/nmol using no-carrier added lutetium-177 ([^177^Lu]LuCl_3_ in 0.04 M HCl; ITM GmbH Medical Isotopes, Munich, Germany) at standard labeling conditions of pH 4.5 as previously reported (Supplementary Material) [21]. After quality control using HPLC, the radiopeptides were used for preclinical investigations without further purification. l-Ascorbic acid (3 mg in 20 µL Milli-Q water) together with sodium acetate (0.5 M, 30 µL in Milli-Q water) were added to the labeling solution after incubation. The radiolytic stability of the radiopeptides at a molar activity of 50 MBq/nmol was assessed in saline at an activity concentration of 150 MBq/300 µL as previously reported (Supplementary Material) [21].

### Blood plasma stability and n-octanol/PBS distribution coefficients

The ^177^Lu-labeled peptides (50 MBq/nmol) were incubated in mouse or human blood plasma (10 MBq/200 µL) over an incubation period of 24 h at 37 °C. Aliquots and control samples of the radiopeptides diluted in saline were analyzed after 1 h, 4 h and 24 h using thin layer chromatography (TLC) as previously reported (Supplementary Material) [21]. The distribution coefficients (logD values) of the ^177^Lu-labeled peptides (50 MBq/nmol) were determined using a shake-flask method according to a previously published procedure (Supplementary Material) [21].

### Albumin-binding properties

The albumin-binding properties of the radiopeptides in mouse and human blood plasma were determined using an ultrafiltration assay with Amicon centrifugal filters (cut-off of 10 kDa; Merck Millipore) as previously reported (Supplementary Material) [21]. A fixed amount of the respective radiopeptide (~ 300 kBq, 15 µL, 6 pmol) was added to a defined volume (150 µL) of mouse and human blood plasma dilutions to obtain [mouse serum albumin]-to-[radiopeptide] molar concentration ratios of 0.01‒12,500 and [human serum albumin]-to-[radiopeptide] molar concentration ratios of 0.01‒20,000. The data were analyzed using a semi-logarithmic Hill plot with the maximum binding set as 100% using GraphPad Prism software (version 10.1.1). The half-maximum binding (B_50_ values) of each radiopeptide was expressed as relative values using the inverse ratios with the value of [^177^Lu]Lu-uPAR-11 set as 1.0.

### Cell uptake and internalization

Human embryonic kidney (HEK) cells transfected with human uPAR, herein referred to as HEK-uPAR cells, were obtained from Innoprot, Innovative Technologies in Biological Systems S.L. (Bizkaia, Spain). Cell uptake and internalization studies of the radiopeptides (50 MBq/nmol) were performed as previously reported [21]. In brief, the HEK-uPAR cells were grown to confluency over night, followed by incubation with the ^177^Lu-labeled peptides (38 kBq, 0.75 pmol per well). An excess of AE105 (5 µM) was used to block uPAR in some of the samples. After incubation of the HEK-uPAR cells with the respective radiopeptide for 2–4 h at 37 °C, the supernatant was removed and the cells were rinsed with ice-cold phosphate buffered saline (PBS) to determine the total uptake of the radiopeptides. HEK-uPAR cells were additionally rinsed with stripping buffer (pH 2.8, 50 mM glycine, 100 mM NaCl) to determine the internalized fraction of the radiopeptides. Afterward, the HEK-uPAR cells were lysed using NaOH solution (1 M, 1 mL), transferred to radioimmunoassay tubes and counted for the activity using a γ-counter (Wallac Wizard 1480, PerkinElmer). The protein concentration of each sample was determined to standardize the measured activity in cells of each well. The uptake and internalized fraction of the radiopeptides were expressed as the percentage of total added activity as average ± SD of *n* = 3 independent experiments.

### uPAR-binding affinity (K_D_ values)

To determine the uPAR-binding affinity of the radiopeptides, saturation binding experiments were performed according to a previously published procedure using HEK-uPAR cells in 48-well plates (Supplementary Material) [21]. The assay was performed on ice (at 4 °C) to prevent target internalization upon binding of the radiopeptides. The cells were incubated with the radiopeptides in the presence or absence of excess AE105 (40 µM) together with the respective radiopeptides at concentrations ranging between 1 nM and 1600 nM. After removal of the supernatant, the HEK-uPAR cells were rinsed and lysed to be measured using a γ-counter (Wallac Wizard 1480, PerkinElmer). The K_D_ values were determined by plotting the specific binding (values of cell samples incubated without AE105 minus the respective values of samples incubated with AE105) against the molar concentration of the added peptide. A nonlinear regression analysis was performed using GraphPad Prism software (version 10.1.1). The results are reported as average of K_D_ values ± SD of *n* = 3 independent experiments.

### Animal experiments

All applicable international, national, and/or institutional guidelines for the care and use of laboratory mice were followed and the studies were carried out according to the guidelines of the Swiss Regulations for Animal Welfare. The experiments were ethically approved by the Cantonal Committee of Animal Experimentation and permitted by the responsible cantonal authorities (License N° 75721). Five-week-old female CD1 nude (Crl: CD1-*Foxn*^*nu*^) and female immunocompetent FVB (FVB/NCrl) mice were obtained from Charles River Laboratories (Sulzfeld, Germany) and fed with standard rodent chow ad libitum. The mice were subcutaneously inoculated with HEK-uPAR cells (7 × 10^6^ cells in 100 µL PBS) on the right shoulder. The growth of the HEK-uPAR cells to xenografts lasted for 3‒9 weeks and varied from mouse to mouse, resulting in xenografts of sizes between 100 mm^3^ and 600 mm^3^. The immunocompetent FVB mice were used for testing the in vivo stability of [^177^Lu]Lu-uPAR-11.

### SPECT/CT imaging studies

Single-photon emission computed tomography/computed tomography (SPECT/CT) experiments were performed using a small-animal SPECT/CT camera (NanoSPECT/CT™, Mediso Medical Imaging Systems, Budapest, Hungary) as described previously (Supplementary Material) [21]. HEK-uPAR xenografted nude mice were scanned at 1 h, 4 h, 24 h and 48 h after injection of the respective radiopeptide (25 MBq, 0.5 nmol, in 100 µL 0.9% NaCl containing 0.05% bovine serum albumin (BSA)) The images were prepared using VivoQuant post-processing software (version 3.5, inviCRO Imaging Services and Software, Boston, USA). A Gauss post-reconstruction filter (full width at half maximum, 1.0 mm) was applied and the scale of activity was set as indicated on the images (minimum value = 0.2 Bq/voxel, maximum value = 20 Bq/voxel).

### Biodistribution studies

HEK-uPAR xenografted mice were injected with the respective radiopeptide (5 MBq, 0.5 nmol/mouse, 100 µL 0.9% NaCl containing 0.05% BSA) and sacrificed at 4 h and 24 h after injection. In the case of [^177^Lu]Lu-uPAR-11, more timepoints were investigated and mice were sacrificed after 1 h, 4 h, 24 h and 48 h post injection (p.i.) for comparison with [^177^Lu]Lu-DOTA-AE105 which was also investigated at 1 h, 4 h and 24 h p.i. The tissues and organs of interest were collected, weighed and counted for activity using a γ-counter (Wallac Wizard 1480, PerkinElmer). The results were reported as the percentage of the injected activity per gram of tissue mass (% IA/g) using standards of the injection solution measured at the same time to obtain decay-corrected data. The results were presented as the average ± SD of *n* = 3 mice. The time-activity curves were determined for the uptake of [^177^Lu]Lu-DOTA-AE105 and [^177^Lu]Lu-uPAR-11 in the blood, HEK-uPAR xenografts, kidneys and liver based on non-decay corrected biodistribution data. For the blood, the initial uptake (t = 0) was set as 50% IA/g assuming a blood volume of 2 mL for CD1 nude mice. The initial uptake value (t = 0) for all other organs and tissues was set as 0% IA/g. The non-decay corrected uptake of [^177^Lu]Lu-DOTA-AE105 at 48 h p.i. was estimated based on the assumption that only the physical decay would be relevant. The area under the curves over 48 h after injection (AUC_0h→48 h_) was calculated using GraphPad Prism software (version 10.1.1). Statistical analyses were performed by applying a one-way ANOVA test with a Dunnet’s multiple comparison post‐test to compare the data of the new radiopeptides with those of [^177^Lu]Lu-DOTA-AE105 and a Tukey’s multiple comparisons post‐test to compare the novel radiopeptides among them. A *p*-value of < 0.05 was considered a statistically significant difference using GraphPad Prism software (version 10.1.1).

### In vivo stability studies

[^177^Lu]Lu-uPAR-11 (50 MBq/nmol) was administered to immunocompetent FVB mice (25 MBq, 0.5 nmol, 100 µL) followed by the collection of urine 1–4 h later to investigate its in vivo stability. After urine sampling, the mice were euthanized (at 1–4 h p.i.) followed by blood sampling from the heart and collection of the liver and kidneys. Blood plasma and urine samples were analyzed to determine the percentage of intact radiopeptide and potential formation of radiometabolites using TLC as described for testing their in vitro stability (see above). Liver and kidney samples were processed as previously reported and analyzed using the same TLC system as for blood plasma and urine samples (Supplementary Material) [21]. The analysis of the thin layer chromatograms was performed as described for the in vitro blood plasma stability experiments.

### Computational modeling

The previously employed computer-based molecular model visualizing the binding of Lu-uPAR-02 in the groove of uPAR was used to model the fit of Lu-uPAR-11, as the structural analogue Lu-uPAR-02 only differed in the albumin-binding entity (Supplementary Material) [19].

## Results

### Synthesis of the uPAR-targeting peptides

The uPAR-targeting peptides were obtained in a 3–13% overall yield after 25 synthetic steps. The purity of the final products was ≥ 98%, as determined by UV-Vis HPLC analyses (Supplementary Material, Fig. S2, Table S2). The mass-to-charge (m/z) ratio calculated for the respective compounds correlated well with experimental HRMS data, confirming the chemical identity of the produced peptides (Supplementary Material, Table S2, Fig. S3‒S8).

### Radiolabeling and radiolytic stability

The preparation of the radiopeptides was feasible at molar activities up to 50 MBq/nmol with a radiochemical purity of ≥ 95% (Supplementary Material, Fig. S9). In the presence of l-ascorbic acid, the radiopeptides were stable with ≥ 91% intact radiopeptide detected after an incubation period of 24 h at room temperature (Table 1, Supplementary Materials, Table S3).

**Table 1:** In vitro data of the radiopeptides, reported as the average ± SD of *n* = 3 independent experiments

Radiopeptide	Radiolytic stability after 24 h incubation	Mouse blood plasma stability after 4 h incubation	Albumin-binding properties^a^	*n*-Octanol/PBS distribution coefficient	uPAR-binding affinity
	Intact radiopeptide [%]	Intact radiopeptide [%]	Relative values	-	[nM]
[^177^Lu]Lu-DOTA-AE105^b^	96 ± 2	13 ± 7	0.06	−1.38 ± 0.18	20 ± 1
[^177^Lu]Lu-uPAR-11	99 ± 1	99 ± 1	1.00^c^	−0.26 ± 0.09	37 ± 11
[^177^Lu]Lu-uPAR-12	96 ± 2	99 ± 1	1.99	0.96 ± 0.15	38 ± 9
[^177^Lu]Lu-uPAR-14	91 ± 5	97 ± 4	0.66	−0.13 ± 0.08	37 ± 7
[^177^Lu]Lu-uPAR-15	91 ± 5	99 ± 1	0.77	−0.03 ± 0.01	42 ± 3
[^177^Lu]Lu-uPAR-17	95 ± 3	97 ± 2	0.77	−1.56 ± 0.03	31 ± 6
[^177^Lu]Lu-uPAR-18	96 ± 6	99 ± 2	0.66	−0.50 ± 0.07	33 ± 4

^a^Values indicated for the binding to albumin in mouse blood plasma; ^b^data were previously published by Beyer et al. 2025, Mol Pharm 22:3242 [21], Copyright 2025 American Chemical Society; ^c^The albumin-binding affinity was arbitrarily set as 1.00

### Blood plasma stability and n-octanol/PBS distribution coefficients

In mouse and human blood plasma ≥ 97% of the respective radiopeptides were found intact after an incubation period of 4 h (Table 1, Supplementary Materials, Tables S4/S5). After a 24-h incubation period in mouse blood plasma, only 61 ± 9% intact [^177^Lu]Lu-uPAR-17 was observed, whereas 96 ± 1% of this radiopeptide were still intact after the same incubation period in human blood plasma. All other radiopeptides showed less degradation in mouse plasma with at least 89% intact radiopeptide detected after 24 h incubation, while at least 95% of intact radiopeptides were detected in human blood plasma over this same period (Supplementary Materials, Tables S4/S5). The distribution coefficients (logD values) determined for the radiopeptides were in the range of −1.56–0.96. (Table 1).

### Albumin-binding properties

**Fig. 2:**
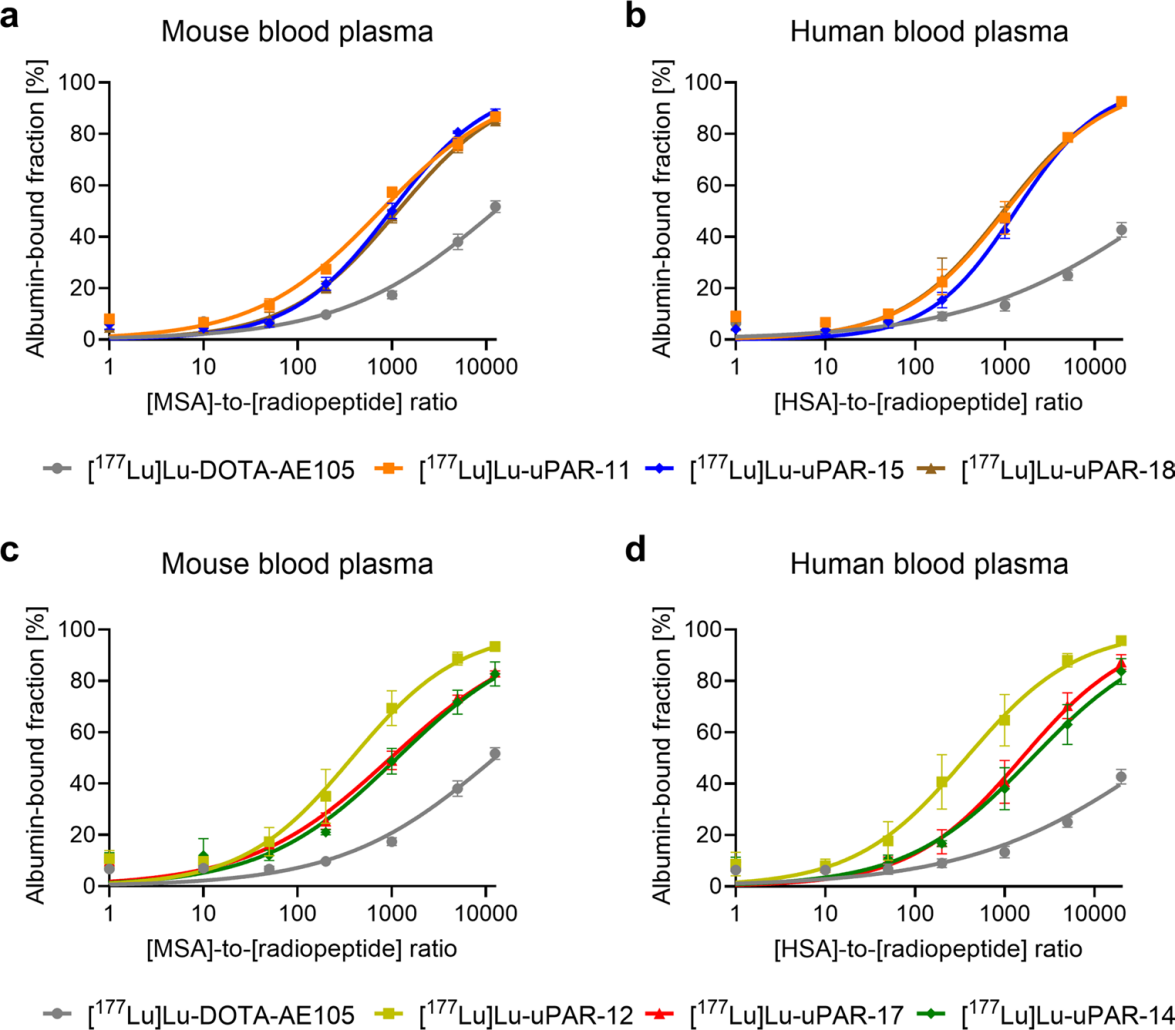
**a-d** Albumin-binding properties of the new radiopeptides compared to those of [^177^Lu]Lu-DOTA-AE105. (**a/c**) Plasma protein-binding curves of the radiopeptides in mouse blood plasma (*n* = 3), (**b/d**) plasma protein-binding curves of the radiopeptides in human blood plasma (*n* = 3)

The new radiopeptides showed 11-fold to 155-fold increased albumin-binding affinity as compared to [^177^Lu]Lu-DOTA-AE105. [^177^Lu]Lu-uPAR-12 showed a 2.0-fold and 2.7-fold increased affinity to mouse and human albumin, respectively, as compared to that of [^177^Lu]Lu-uPAR-11, while [^177^Lu]Lu-uPAR-14, [^177^Lu]Lu-uPAR-15 and [^177^Lu]Lu-uPAR-17 had a 0.50‒0.77-fold weaker albumin-binding affinity than [^177^Lu]Lu-uPAR-11. [^177^Lu]Lu-uPAR-18 showed a 0.66-fold weaker binding to mouse albumin, but almost identical (1.05-fold increased) affinity to human albumin as compared to that of [^177^Lu]Lu-uPAR-11 (Fig. 2; Table 1, Supplementary Materials, Table S6).

### Cell uptake of the radiopeptides

**Fig. 3:**
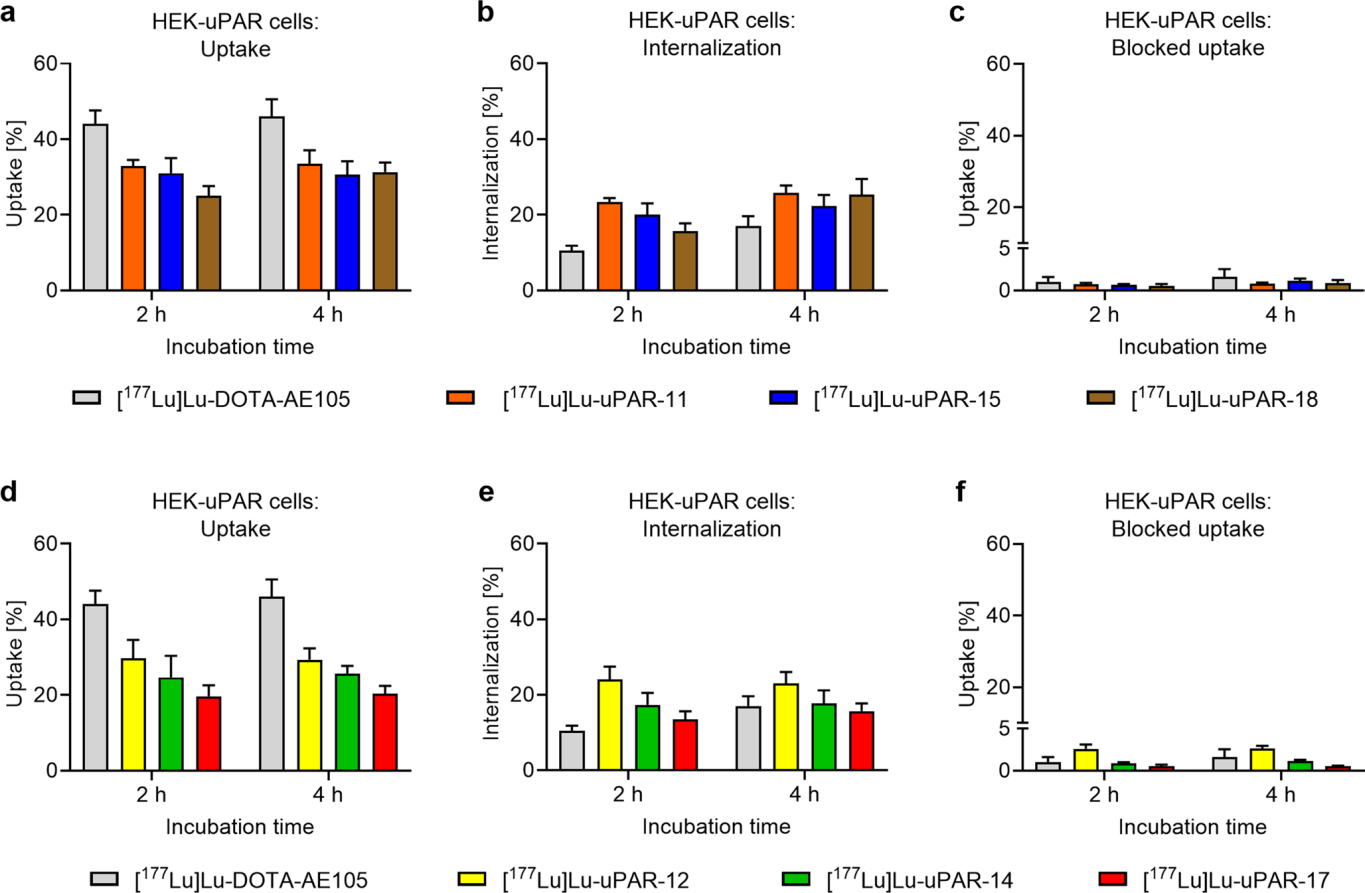
**a-f** Cell uptake with and without co-incubation of AE105 to block uPAR and internalization of the radiopeptides in HEK-uPAR cells after 2 h and 4 h incubation at 37 °C. (**a**/**d**) Cell uptake of the radiopeptides, (**b/e**) cell internalized fraction of the radiopeptides, (**c/f**) blocked uptake of the radiopeptides

The uptake of radiopeptides in HEK-uPAR cells reached 20‒32% and 20‒34% of total added activity over an incubation period of 2 h and 4 h, respectively (Fig. 3a/d). The data obtained for [^177^Lu]Lu-DOTA-AE105 showed an uptake of 44 ± 3% and 46 ± 5% after 2 h and 4 h, respectively. The albumin-binding radiopeptides showed an internalized fraction of 16–24% and 16–26% of total added activity after 2 h and 4 h (Fig. 3b/e), respectively, whereas that of [^177^Lu]Lu-DOTA-AE105 was 11 ± 1% and 17 ± 2% after 2 h and 4 h, respectively [21]. The uptake of all radiopeptides was blocked by an excess of added AE105, resulting in negligible uptake of 0.5–2.6% and 0.5–2.6% after 2 h and 4 h, respectively (Fig. 3c/f).

### uPAR-binding affinity of the radiopeptides

The K_D_ values of all radiopeptides were in the range of 31‒42 nM which was similar to the K_D_ value of 20 ± 1 nM determined for [^177^Lu]Lu-DOTA-AE105. Among the new uPAR-targeting radiopeptides, [^177^Lu]Lu-uPAR-17 showed the highest uPAR-binding affinity (K_D_ value 31 ± 6 nM) and [^177^Lu]Lu-uPAR-15 the lowest (K_D_ value 42 ± 3 nM) (Table 1, Supplementary Material, Fig. S10).

### SPECT imaging studies

**Fig. 4:**
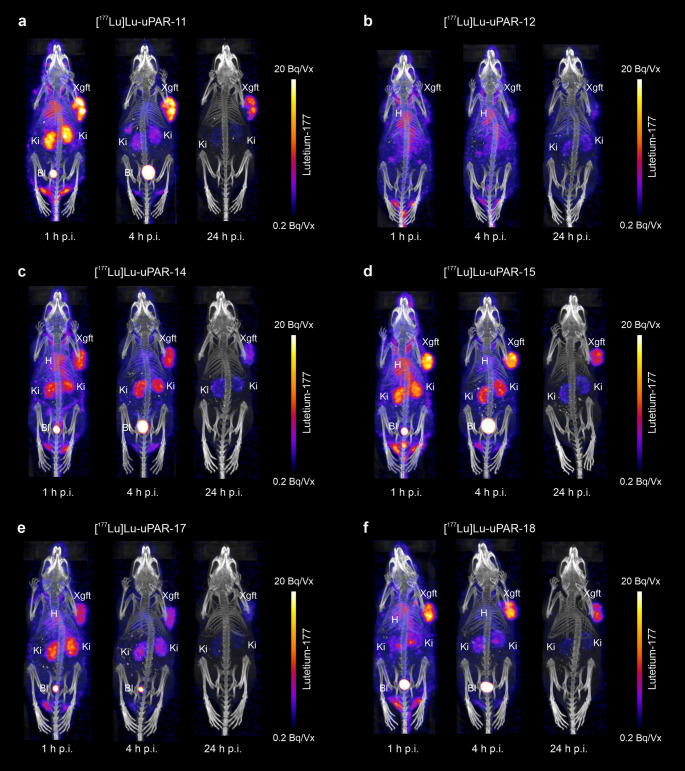
**a-f** SPECT/CT images of HEK-uPAR-xenografted mice acquired at 1 h, 4 h and 24 h p.i. after injection of the respective radiopeptide. (**a**) [^177^Lu]Lu-uPAR-11, (**b**) [^177^Lu]Lu-uPAR-12, (**c**) [^177^Lu]Lu-uPAR-14, (**d**) [^177^Lu]Lu-uPAR-15, (**e**) [^177^Lu]Lu-uPAR-17 and (**f**) [^177^Lu]Lu-uPAR-18. Ki = kidney, Bl = urinary bladder, Xgrft = HEK-uPAR xenograft, H = heart

The SPECT/CT images showed considerable retention of activity in the blood and heart 1 h after injection of the radiopeptides (Fig. 4a-f). Most of the radiopeptides were, however, effectively cleared from the blood over the following hours so that the signal in the heart was almost entirely gone at 4 h p.i. in all cases except in mice injected with [^177^Lu]Lu-uPAR-12 (Fig. 4b). In this case, substantial blood retention was observed over the entire time of investigation. The accumulation of [^177^Lu]Lu-uPAR-11, [^177^Lu]Lu-uPAR-15 and [^177^Lu]Lu-uPAR-18 in the HEK-uPAR xenografts was highest at 1 h p.i. and relatively well retained over the following hours (Fig. 4a/d/f). Substantial activity in the xenografts was also observed over the first 4 h after injection of [^177^Lu]Lu-uPAR-14 and [^177^Lu]Lu-uPAR-17, but in these cases, it was almost entirely cleared at 24 h p.i. (Fig. 4c/e). The xenograft uptake of [^177^Lu]Lu-uPAR-12 was only moderate over the entire period of investigation (Fig. 4b).

All radiopeptides showed substantial accumulation in the kidneys 1 h after injection, with the only exception of [^177^Lu]Lu-uPAR-12. Renal clearance varied for the single radiopeptides with some showing only a faint signal in the kidneys at 4 h p.i. (Fig. 4a/e/f) while others still showed substantial renal uptake at this same timepoint (Fig. 4c/d). All radiopeptides were, however, almost entirely cleared from the kidneys after one day as demonstrated by the absence of signal on the 24 h p.i. SPECT scans.

### Biodistribution studies

**Fig. 5:**
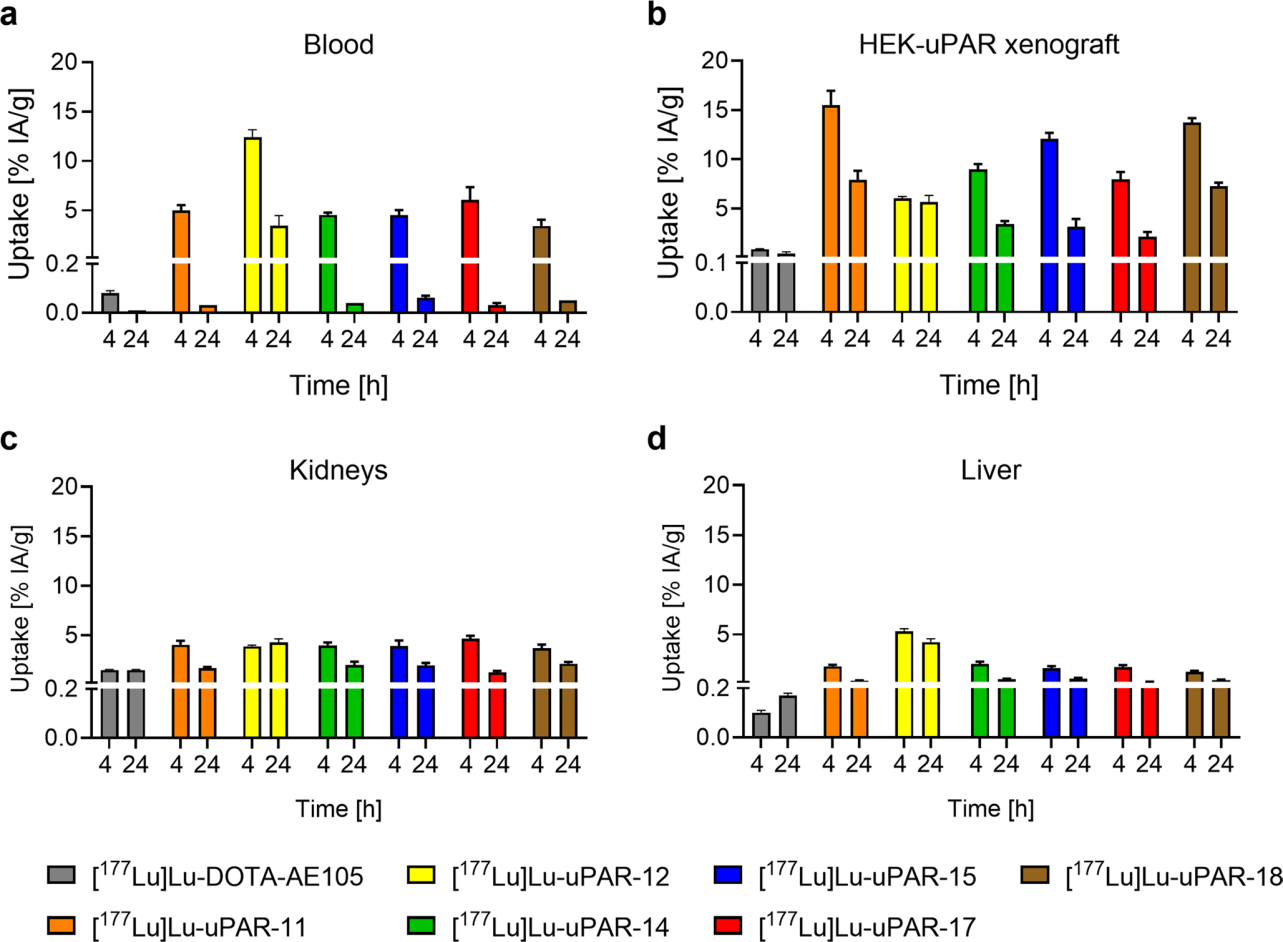
**a-d** Biodistribution data of HEK-uPAR xenograft-bearing nude mice 4 h and 24 h after injection of [^177^Lu]Lu-uPAR-11, [^177^Lu]Lu-uPAR-12, [^177^Lu]Lu-uPAR-14, [^177^Lu]Lu-uPAR-15, [^177^Lu]Lu-uPAR-17 and [^177^Lu]Lu-uPAR-18 compared to that of [^177^Lu]Lu-DOTA-AE105 (adapted with permission from Beyer *et* al. 2025, Mol Pharm 22:3242 [21]. Copyright 2025 American Chemical Society). (**a**) Uptake of the radiopeptides in blood, (**b**) uptake of the radiopeptides in HEK-uPAR xenograft, (**c**) uptake of the radiopeptides in the kidneys, (**d**) uptake of the radiopeptides in the liver

Biodistribution data of the new uPAR-targeting radiopeptides were investigated and compared with those obtained for [^177^Lu]Lu-DOTA-AE105 (Fig. 5; Supplementary Material, Table S7‒13). [^177^Lu]Lu-DOTA-AE105 was completely cleared from the blood circulation at 4 h p.i. (0.08 ± 0.01% IA/g) (Fig. 5a), while the new radiopeptides showed still 3.4–12% IA/g blood activity at this same timepoint (*p* < 0.05). After 24 h, most of the new radiopeptides were also cleared entirely from the blood. [^177^Lu]Lu-uPAR-12 was the only exception, which showed still 3.4 ± 1.0 IA/g blood activity at 24 h p.i.. Comparison of the blood retention among the new radiopeptides revealed a similar clearance for all compounds (*p >* 0.05) except for [^177^Lu]Lu-uPAR-12 which showed a significantly slower blood clearance (*p <* 0.05).

At 4 h after injection of the new radiopeptides, the uptake in the HEK-uPAR xenografts reached 6.0–16% IA/g, which was significantly higher than 0.87 ± 0.05% IA/g uptake reached after injection of [^177^Lu]Lu-DOTA-AE105 (*p <* 0.05). The tumor uptake of the new radiopeptides at 24 h p.i. ranged from 2.1% IA/g to 7.9% IA/g while the tumor accumulation of [^177^Lu]Lu-DOTA-AE105 was only 0.40 ± 0.19% IA/g (Fig. 5b). The comparison among the new radiopeptides revealed that [^177^Lu]Lu-uPAR-11 reached a significantly higher xenograft accumulation of 16 ± 2% IA/g and 7.9 ± 0.9% IA/g at 4 h and 24 h p.i., respectively, compared to all other radiopeptides (*p <* 0.05). The only exception was [^177^Lu]Lu-uPAR-18, which showed a comparable xenograft uptake of 14 ± 1% IA/g and 7.3 ± 0.4% IA/g at 4 h and 24 h, respectively, as found for [^177^Lu]Lu-uPAR-11 (*p >* 0.05).

Kidney retention of the novel radiopeptides was in the range of 3.7‒4.6% IA/g at 4 h p.i., which was significantly higher (*p <* 0.05) than the 1.5 ± 0.1% IA/g observed for [^177^Lu]Lu-DOTA-AE105. At 24 h after injection of [^177^Lu]Lu-uPAR-11 and [^177^Lu]Lu-uPAR-17, kidney retention reached levels between 1.3% IA/g and 1.7% IA/g, similar to that of [^177^Lu]Lu-DOTA-AE105 (1.3 ± 0.1% IA/g; *p >* 0.05). In contrast, all other radiopeptides were more retained with 2.0–4.3% IA/g uptake in the kidneys at that timepoint (Fig. 5c). The liver accumulation of all new radiopeptides was higher than for [^177^Lu]Lu-DOTA-AE105 at 4 h p.i. (*p <* 0.05). After 24 h p.i., these values were, however, similar (*p >* 0.05), except for [^177^Lu]Lu-uPAR-12, which still exhibited a significantly higher liver accumulation than [^177^Lu]Lu-DOTA-AE105 (*p <* 0.05). The liver uptake of the novel radiopeptides was < 2.0% IA/g and < 0.5% IA/g at 4 h and 24 h p.i., respectively, only [^177^Lu]Lu-uPAR-12 showed a significantly higher accumulation of 5.3 ± 0.3% IA/g and 4.2 ± 0.3% IA/g at 4 h and 24 h, respectively (*p <* 0.05). (Fig. 5d).

**Fig. 6:**
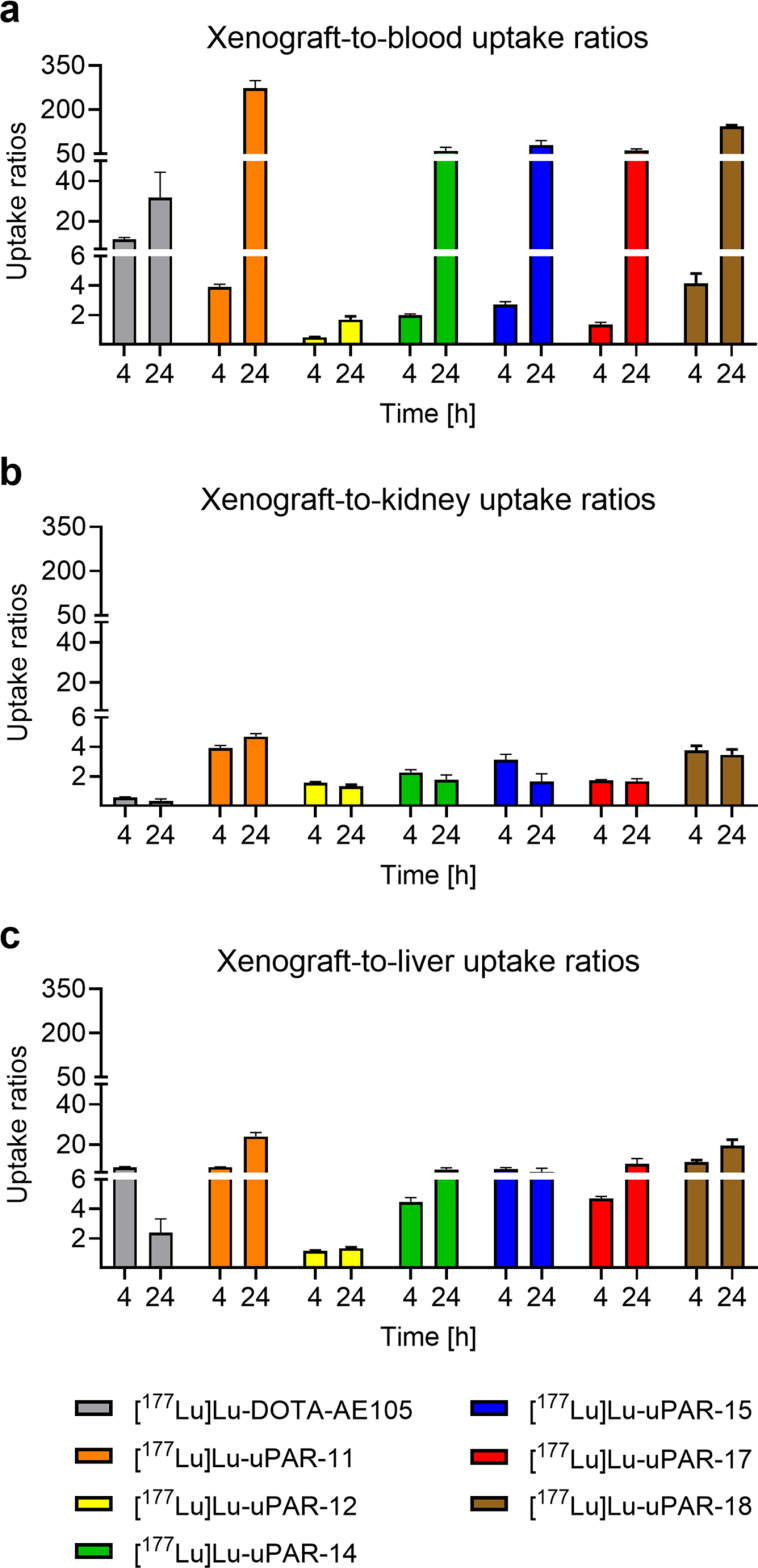
**a-c** Xenograft-to-background ratios of accumulated activity in HEK-uPAR xenografted mice at 4 h and 24 h after injection of the uPAR-targeting radiopeptides. (**a**) Xenograft-to-blood uptake ratios, (**b**) xenograft-to-kidney uptake ratios, (**c**) xenograft-to-liver uptake ratios

At 4 h p.i. the xenograft-to-blood ratio of accumulated [^177^Lu]Lu-DOTA-AE105 reached 11 ± 1 while those of the new radiopeptides ranged between 0.49 and 4.1 (*p <* 0.05). After 24 h p.i., the new radiopeptides showed xenograft-to-blood uptake ratios ranging from 61 to 274 which was higher than for [^177^Lu]Lu-DOTA-AE105 (32 ± 13). An exception remained [^177^Lu]Lu-uPAR-12 which exhibited a tumor-to-blood uptake ratio of 1.7 ± 0.2 at this timepoint (Fig. 6a). The xenograft-to-kidney uptake ratios of the novel radiopeptides reached 1.6–3.9 and 1.3–4.7 at 4 h and 24 h after injection, respectively, while the same ratios of [^177^Lu]Lu-DOTA-AE105 reached only 0.58 ± 0.03 and 0.33 ± 0.14, respectively (Fig. 6b). The xenograft-to-liver uptake ratios of [^177^Lu]Lu-uPAR-12, [^177^Lu]Lu-uPAR-14 and [^177^Lu]Lu-uPAR-17 were significantly lower than the uptake ratio of [^177^Lu]Lu-DOTA-AE105 (*p <* 0.05), while the xenograft-to-liver uptake ratios of [^177^Lu]Lu-uPAR-11, [^177^Lu]Lu-uPAR-15 and [^177^Lu]Lu-uPAR-18 were similar or even higher at 4 h p.i.

**Fig. 7:**
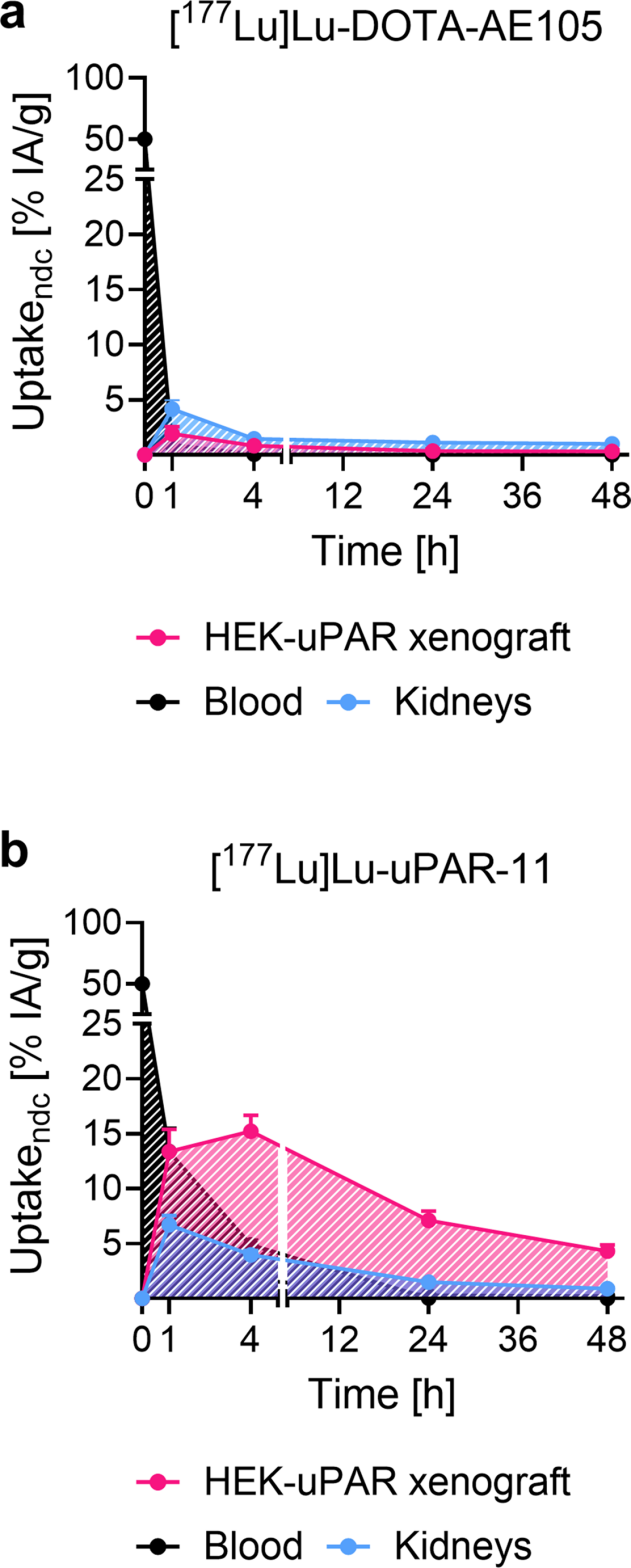
**a/b** Time-activity curves for the uptake of the radiopeptides in the blood, in HEK-uPAR xenografts and in the kidneys shown as areas under the curve (AUC). (**a**) AUC_0h→48 h_ after injection of [^177^Lu]Lu-DOTA-AE105 whereof the 48-h timepoint was extrapolated based on the decay only, (**b**) AUC_0h→48 h_ after injection of [^177^Lu]Lu-uPAR-11

The biodistribution of [¹⁷⁷Lu]Lu-uPAR-11 was further evaluated 1 h and 48 h p.i. to enable time-activity curves and determine the AUC_0h→48 h_ values for various organs and facilitate comparison with [¹⁷⁷Lu]Lu-DOTA-AE105 (Fig. 7; Supplementary Material, Table S14). The time activity curves for the blood uptake resulted in a 3.9-fold increased AUC_0h→48 h_ for [¹⁷⁷Lu]Lu-uPAR-11 than for [¹⁷⁷Lu]Lu-DOTA-AE105. On the other hand, the AUC_0h→48 h_ of the time-activity curve for the HEK-uPAR xenograft after application of [¹⁷⁷Lu]Lu-uPAR-11 was 16-fold larger than that of [¹⁷⁷Lu]Lu-DOTA-AE105. This means that the xenograft-to-blood AUC_0h→48 h_ ratio of [¹⁷⁷Lu]Lu-uPAR-11 was 4.2-fold higher than that of [¹⁷⁷Lu]Lu-DOTA-AE105. The time activity curves for kidneys and liver resulted in ~ 1.7-fold and ~ 5.5-fold increased AUC_0h→48 h_ values for [¹⁷⁷Lu]Lu-uPAR-11 than for [¹⁷⁷Lu]Lu-DOTA-AE105.

### In vivo stability of the radiopeptides

Blood plasma, liver, kidneys and urine samples were analyzed to assess the presence of radiometabolites following the injection of [^177^Lu]Lu-uPAR-11 and compared to previously published data obtained for [^177^Lu]Lu**-**DOTA-AE105 [21]. The results revealed metabolization of [^177^Lu]Lu-uPAR-11 as early as 1 h p.i. in blood plasma resulting in 66% intact radiopeptide. In kidney tissue, 19% intact radiopeptide was detected, while it was 32% in the liver. At 4 h after injection of [^177^Lu]Lu-uPAR-11, the intact fraction was lower in all probes (34% intact radiopeptide in plasma, 17% in liver and 11% in kidneys). The analysis of urine samples after 1 h and 4 h indicated that the radiopeptide was almost entirely excreted in metabolized form (1.9–4.5% intact radiopeptide) (Supplementary Material, Table S15).

### Computational modeling

The computer model showed unhindered binding of uPAR-11 irrespective of the conjugated albumin binder. It also indicated that variations in linker length, macrocyclic chelator, or albumin binder do not significantly affect uPAR association, as these moieties do not engage in direct interactions with the receptor (Supplementary Material, Fig. S11).

## Discussion

The herein developed radiopeptides with a *p*-tolyl entity exhibited uPAR-binding affinities and in vitro cell uptake levels in the same range as those of the previous generation uPAR-targeting radiopeptides modified with a *p*-iodophenyl entity and those of [^177^Lu]Lu-DOTA-AE105 [21]. This was also expected based on the similarities in structure and the computational model previously established to fit Lu-uPAR-02 and now adapted for Lu-uPAR-11 [21]. The new radiopeptides showed a shift to weaker albumin-binding affinities as compared to those of the previous generation [21], which was anticipated based on our previous work with PSMA radioligands [23]. Indeed, [^177^Lu]Lu-uPAR-11 comprising a *p*-tolyl entity showed a 3.2-fold and 4.6-fold weaker affinity to mouse and human albumin, respectively, than the *p*-iodophenyl-based analogue [^177^Lu]Lu-uPAR-02 [21]. As a result, the in vivo pharmacokinetic profile of [^177^Lu]Lu-uPAR-11 was substantially improved with ~ 1.5-fold and ~ 1.3-fold higher xenograft accumulation at 4 h p.i. and 24 h p.i., respectively, compared to that of [^177^Lu]Lu-uPAR-02. Of note, all of the herein described radiopeptides with a *p*-tolyl-based albumin binder showed higher xenograft-to-organ ratios of accumulated activity than was the case for the longer-circulating *p*-iodophenyl-based radiopeptides.

Variations in the PEG spacer next to the *p*-tolyl entity in [^177^Lu]Lu-uPAR-15 and [^177^Lu]Lu-uPAR-18 showed albumin-binding affinities in a similar range as that of [^177^Lu]Lu-uPAR-11 in both mouse and human blood plasma. In contrast, [^177^Lu]Lu-uPAR-12 with an aliphatic linker instead of the PEG spacer showed a 2.0-fold and 2.7-fold stronger binding to mouse and human albumin, respectively. These findings were in agreement with previous studies reported by Siwowska et al., who found that the lipophilic aliphatic spacer in close proximity to the albumin-binder of a folate radioconjugate enhanced the albumin-binding affinity, while a PEG spacer reduced the affinity [24]. Overall, [^177^Lu]Lu-uPAR-11 exhibited a marginally favorable pharmacokinetic profile than [^177^Lu]Lu-uPAR-15 and similar characteristics to [^177^Lu]Lu-uPAR-18. The variable degree of hydrophilicity also had an impact on the tissue distribution profile of the radiopeptides. This was best demonstrated by the exchange of the DOTA chelator with a DOTAGA chelator in [^177^Lu]Lu-uPAR-17, which increased the retention of the radiopeptide in the kidneys considerably. This finding may be ascribed to the presence of the additional negative charge of the non-coordinated carboxyl group. This ultimately resulted in a less favorable xenograft-to-kidney ratio of accumulated activity after injection of [^177^Lu]Lu-uPAR-17. The same phenomenon was previously reported for [^177^Lu]Lu-PSMA-I&T, in which the DOTAGA chelator obviously triggered the uptake and retention in the kidneys of mice as compared to the situation after injection of [^177^Lu]Lu-PSMA-617, which comprises a DOTA chelator [25]. As the kidney uptake of [^177^Lu]Lu-PSMA-I&T relative to that of [^177^Lu]Lu-PSMA-617 was only marginally increased in patients [26], the effect of the DOTAGA chelator seen in mice would most probably not be a major concern in the clinical setting.

The herein reported albumin-binding radiopeptides showed improved in vitro plasma stability compared to that of [^177^Lu]Lu-DOTA-AE105, similarly to the uPAR-targeting radiopeptides modified with a *p*-iodophenyl entity [21]. Pronounced degradation was only observed in the case of [^177^Lu]Lu-uPAR-17 when incubated in mouse blood plasma after 24 h, which was, however, not the case after incubation in human blood plasma. Substantial in vivo degradation of [^177^Lu]Lu-uPAR-11 was also observed at 1 h and 4 h after injection of FVB mice (without tumors). Nevertheless, [^177^Lu]Lu-uPAR-11 was still considerably more stable than [^177^Lu]Lu-DOTA-AE105, which was almost entirely degraded in vivo within the first hour after application as previously demonstrated [21]. Obviously, the degradiation of the radiopeptides correlates negatively with the albumin-binding properties, which also explains the even higher stability of [^177^Lu]Lu-uPAR-02 as compared to [^177^Lu]Lu-uPAR-11 due to its stronger albumin-binding properties and, hence, increased albumin-bound fraction [21]. As these in vivo studies were performed in mice without xenografts, it remains unexplored as to whether the metabolism leading to radiopeptide degradation also occured in the HEK-uPAR xenografts. A detailed assessment of the radiopeptide’s metabolism would, thus, be necessary when translating the research to mouse models with human tumor xenografts that reflect the patient situation more accurately.

Common observations from studies with albumin-binding radioligands include the concern for hematopoietic toxicity resulting from increased radiation exposure of the bone marrow [27]. [^177^Lu]Lu-uPAR-11 exhibited a blood residence time in mice that was, however, similar to that of [^177^Lu]Lu-Ibu-DAB-PSMA, which showed no evidence of hematopoietic toxicity in preclinical studies [28]. It remains to be investigated in first-in-human trials, however, as to whether the blood residence time of [^177^Lu]Lu-uPAR-11 would be acceptable, considering the cumulative bone marrow dose limit of 2 Gy [29] and a therapeutic application of several GBq activity per treatment cycle.

A limitation of this study refers to the use of a xenograft mouse model that does not accurately represent the pathological context of uPAR expression on tumor cells or related stromal cells. Nevertheless, this mouse model served for comparison of the radiopeptides, revealing that the xenograft accumulation, in particular of [^177^Lu]Lu-uPAR-11, was considerably higher than that of the parent compound [^177^Lu]Lu-DOTA-AE105. The biodistribution data and xenograft-to-organ uptake ratios of [^177^Lu]Lu-uPAR-11 were superior to those of most of the other herein developed radiopeptides. Only [^177^Lu]Lu-uPAR-18 showed similar data and, hence, future preclinical studies may include both, [^177^Lu]Lu-uPAR-11 and [^177^Lu]Lu-uPAR-18 to evaluate their therapeutic potential in tumor-bearing mice.

## Conclusions

This study demonstrated the advantages of modifying the DOTA-AE105-based radiopeptide with a *p*-tolyl-based albumin-binding entity to extend the blood circulation time of the resultant radiopeptides and, hence, increase xenograft accumulation. Among six radiopeptides, [^177^Lu]Lu-uPAR-11 was identified as the most promising candidate. This radiopeptide should, therefore, be further investigated preclinically in view of its potential for clinical translation and first-in-human application.

## Supplementary Information

Below is the link to the electronic supplementary material.

Supplementary Material 1 (DOCX 2.60 MB)

## Data Availability

All preclinical data analyzed during this study are included in this published article and reported in the Supplementary Information. Additional information or more detailed data are available from the corresponding author on reasonable request.
