# Supplementary material for: Optimizing uPAR-targeting radiopeptides for improved tissue distribution: progress towards radionuclide therapy
Source: Eur J Nucl Med Mol Imaging. 2025 Oct 27;53(4):2502–17. doi: 10.1007/s00259-025-07602-7 (PMC12920294; doi:10.1007/s00259-025-07602-7)
Supplement: Supplementary file 1 — Supplementary Material 1 (DOCX 2.60 MB) [file 259_2025_7602_MOESM1_ESM.docx]

**SUPPLEMENTARY MATERIAL**

**Optimizing uPAR-targeting radiopeptides for improved tissue distribution: Progress towards radionuclide therapy**

Christian Vaccarin^1†^, Darja Beyer^1†^, Jerome V. Schmid^1^, Bastian Klein^1^, Jathursa Jegathasan^1^, Shreshtha Behera^1^, Xavier Deupi^2,3,4^, Roger Schibli^1, 5^ and Cristina Müller^1, *^

1. Center for Radiopharmaceutical Sciences, PSI Center for Life Sciences, 5232 Villigen-PSI, Switzerland

2. Condensed Matter Theory Group, PSI Center for Scientific Computing, Theory, and Data, 5232 Villigen-PSI, Switzerland

3. Laboratory of Biomolecular Research, PSI Center for Life Sciences, 5232 Villigen-PSI, Switzerland

4. Swiss Institute of Bioinformatics (SIB), 1015 Lausanne, Switzerland

5. Department of Chemistry and Applied Biosciences, ETH Zurich, 8093 Zurich, Switzerland.

†Equally contributed

***Correspondence to**:

Prof. Dr. Cristina Müller

Center for Radiopharmaceutical Sciences

PSI Center for Life Sciences

5232 Villigen-PSI

Switzerland

e-mail: cristina.mueller@psi.ch

phone: +41 56 310 44 54

**1. Synthesis of the uPAR-targeting peptides**

***Purpose:*** New uPAR-targeting peptides were designed with the *p*-tolyl entity as a moderate albumin-binding entity. uPAR-11, uPAR-12, uPAR-14, uPAR-15, uPAR-17 and uPAR-18 were synthesized using the methodology of solid-phase peptide synthesis (Fig. S1).


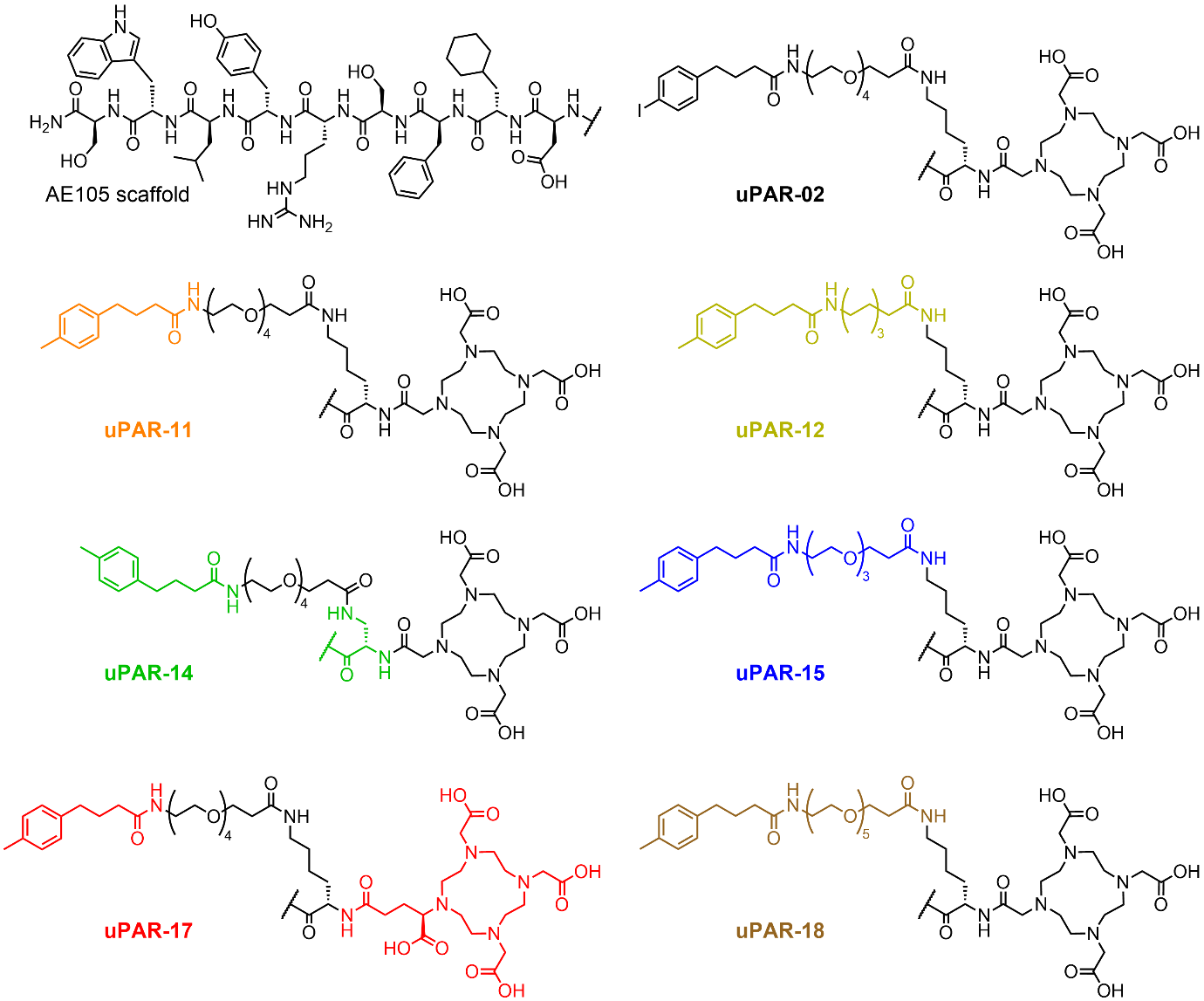


**Fig. S1** Chemical structures of the previously reported uPAR-02 with the *p*-iodophenyl entity as an albumin binder [1] and new uPAR-targeting peptides based on the AE105 peptide, a chelator and a *p*-tolyl entity as an albumin binder

***Methods:*** The synthesis of the new uPAR-targeting peptides was performed by the methodology of solid-phase peptide synthesis according to established synthetic procedures as previously described by Beyer *et al.* [1]. All commercially available solvents and chemicals were used without further purification.

***Synthesis of uPAR-targeting peptides.*** The resin-immobilized and side chains protected AE105 scaffold was synthesized using Rink amide methylbenzhydrylamine resin as solid support. The amino acid residues (0.40 mmol, 4.0 equiv; Fmoc-l-Ser-OH, Fmoc- l-Trp(Boc)-OH, Fmoc-l-Leu-OH, Fmoc-l-Tyr(^t^Bu)-OH, Fmoc-d-Arg(Pbf)-OH, Fmoc-d-Ser(^t^Bu)-OH, Fmoc-l-Phe-OH and Fmoc-l-Cha-OH) were sequentially conjugated using a mixture of *O*-(benzotriazol-1-yl)-*N,N,N',N'*-tetramethyluronium-hexafluorophosphate (HBTU, 0.396 mmol, 3.96 equiv) and *N,N*-diisopropylethylamine (DIPEA, 0.80 mmol, 8.0 equiv) in dimethylformamide (DMF). The coupling of the last amino acid residue (Fmoc-l-Asp(O^t^Bu)-OH), as well as the following coupling steps reported for the preparation of the new uPAR-targeting peptides, used 1-hydroxybenzotriazole hydrate (HOBt, 0.40 mmol, 4.0 equiv) as part of the reaction mixture to suppress unwanted cyclization reactions. The Fmoc deprotection was performed by agitation twice for 5 min using a 1:1 (*v*/*v*) DMF/piperidine solution. After each reaction step, the resin was washed with DMF to remove the remaining reagent residues. The resin-immobilized and side chain-protected peptide scaffold (**1**) served as a starting material for the synthesis of the albumin-binding, uPAR-targeting peptides. uPAR-11 was prepared by conjugation of the Dde-l-Lys(Fmoc)-OH (0.40 mmol, 4.00 equiv) to the resin-immobilized AE105 peptide chain (compound **1**, 0.10 mmol, 1.00 equiv, Scheme S1). After Fmoc deprotection, the *N*_ɛ_ group of the lysine residue was reacted with a 3-[2-[2-[2-[2-(9H-fluoren-9-ylmethoxycarbonylamino)ethoxy]ethoxy]ethoxy]ethoxy] propanoic acid (Fmoc-*N*-amido-PEG_4_-acid) followed by Fmoc deprotection and conjugation of 4-(*p*-tolyl)butanoic acid. The remaining 1-(4,4-dimethyl-2,6-dioxocyclohex-1-ylidene)ethyl (Dde) protecting group of the lysine residue was cleaved using a mixture of 2% (*v/v*) hydrazine hydrate in DMF twice for 30 min before conjugation of 2-[4,7,10-tris[2-[(2-methylpropan-2-yl)oxy]-2-oxoethyl]-1,4,7,10-tetrazacyclododec-1-yl]acetic acid (DOTA-tris(^t^Bu)ester, 0.40 mmol, 4.00 equiv). Cleavage from the resin and general deprotection of the peptide was performed by exposing the intermediate to a trifluoroacetic (TFA) solution containing 2.5% (*v/v*) triisopropylsilane and 2.5% (*v/v*) Milli-Q water for a total of 3 h, yielding the crude uPAR-11. The other uPAR-targeting peptides (uPAR-12, uPAR-14, uPAR-15, uPAR-16 and uPAR-18) were synthesized following a similar approach but adapting the reagent used in the specific reaction steps. uPAR-12 was synthesized by replacing the Fmoc-*N*-amido-PEG_4_-acid with Fmoc-8-(9H-fluoren-9-ylmethoxycarbonylamino)octanoic acid (8-Aoc-OH) in the respective synthesis step. The replacement of Fmoc-*N*-amido-PEG_4_-acid with a shorter 3-[2-[2-[2-(9H-fluoren-9-ylmethoxycarbonylamino)ethoxy]ethoxy]ethoxy]propanoic acid (Fmoc-*N*-amido-PEG_3_-acid) or longer 3-[2-[2-[2-[2-[2-(9H-fluoren-9-ylmethoxycarbonylamino)ethoxy]ethoxy]ethoxy] ethoxy]ethoxy]propanoic acid (Fmoc-*N*-amido-PEG_5_-acid) entity yielded uPAR-15 and uPAR-18, respectively. uPAR-14 was synthesized by replacing the Dde-l-Lys(Fmoc)-OH residue used in the preparation of uPAR-11 with (2*S*)-2-(9H-fluoren-9-ylmethoxycarbonylamino)-3-[1-(2-hydroxy-4,4-dimethyl-6-oxocyclohexen-1-yl)ethylideneamino]propanoic acid (Dde-l-Dap(Fmoc)-OH) as a shorter trifunctional linker in the respective synthesis step. Finally, for the synthesis of uPAR-17, (*R*)-5-[(2-methylpropan-2-yl)oxy]-5-oxo-4-[4,7,10-tris[2-[(2-methylpropan-2-yl)oxy]-2-oxoethyl]-1,4,7,10-tetrazacyclododec-1-yl]pentanoic acid ((*R*)-DOTAGA-tetra(^t^Bu)ester) was coupled instead of DOTA-tris(^t^Bu)ester as was the case for uPAR-11 (Scheme S1).

**Scheme S1**. Representative synthetic pathway pursued for the preparation of the uPAR-targeting peptides; in all cases, a *p*-tolyl entity was used as an albumin-binding entity

***Purification and characterization of the synthesized uPAR-targeting peptides.*** In each case, the crude peptide was dissolved in a 1:1 (*v*/*v*) mixture of acetonitrile (ACN) and Milli-Q water. The subsequent purification was performed using a semipreparative high performance liquid chromatography (HPLC) system (Merck-Hitachi LaChrom system, Darmstadt, Germany; including a D-7000 interface, L-7200 autosampler, L-7400 UV-Vis detector, L-7100 pump) equipped with a reversed-phase C18 column (Sunfire^TM^, 5 μm, 10×150 mm, Waters, Milford, MA, USA). The respective peptide was eluted using variable linear gradients of Milli-Q water containing 0.1% TFA (eluent A) and ACN (eluent B) (Table S1). The desired products were detected by evaluating the eluate absorbance at λ=254 nm. The product-containing fractions were collected and combined in a round bottom flask, frozen in liquid nitrogen and lyophilized overnight. The chemical identity of the final products was confirmed by high-resolution mass spectrometry (HRMS) analysis (MALDI-TOF-MS, Bruker UltraFlex II, Billarica, MA, USA).

**Table S1** HPLC elution conditions used for the purification of the uPAR-targeting peptides

| **Compound** | **Gradient**  **[% H_2_O]** | **Gradient**  **[% ACN]** | **Flow rate**  **[mL/min]** | **Run time**  **[min]** | **t_R_**  **[min]^a^** |
| --- | --- | --- | --- | --- | --- |
| uPAR-11 | 75‒55 | 25‒45 | 2 | 20 | 17.1‒18.1 |
| uPAR-12 | 75‒50 | 25‒50 | 2 | 20 | 17.0‒17.6 |
| uPAR-14 | 65‒60 | 35‒40 | 2 | 20 | 10.2‒11.8 |
| uPAR-15 | 65‒60 | 35‒40 | 2 | 25 | 11.7‒13.0 |
| uPAR-17 | 75‒50 | 25‒50 | 2 | 20 | 13.2‒14.2 |
| uPAR-18 | 70‒68 | 30‒32 | 2 | 20 | 16.5‒18.0 |

^a^ Retention time (t_R_) range of the collected fractions containing the products of interest

The chemical purity of the final products was determined by HPLC using the same system as described above, equipped with an analytical reversed-phase C18 column (Sunfire^TM^, 5 μm, 4.6×150 mm, Waters, Milford, MA, USA). The peptides were eluted using a linear gradient of Milli-Q water containing 0.1% TFA (95–20%) and ACN (5–80%) over 15 min at a flow rate of 1 mL/min and subsequently detected by determining their absorbance at λ = 254 nm.

***Results:*** The peptides were obtained with ≥98% chemical purity but moderate overall yields of 3–13% (Fig. S2). The measured HRMS data and their correlation with theoretical values are listed in Table S2 and shown in Figs. S3-S8.

**Table S2** Chemical characterization data of the uPAR-targeting peptides

| **Compound** | **adduct ion** | **m/z_calc_** | **m/z_found_** ^a^ | **Yield** | **Purity** ^b^ |
| --- | --- | --- | --- | --- | --- |
| uPAR-11 | [M+H]^+^ | 2147.1423 | 2147.1401 | 12% | >98% |
| uPAR-12 | [M+H]^+^ | 2041.1157 | 2041.1165 | 11% | >98% |
| uPAR-14 | [M+H]^+^ | 2105.0954 | 2105.0968 | 3% | >98% |
| uPAR-15 | [M+H]^+^ | 2103.1161 | 2103.1161 | 10% | >98% |
| uPAR-17 | [M+H]^+^ | 2219.1634 | 2219.1637 | 8% | 98% |
| uPAR-18 | [M+H]^+^ | 2191.1685 | 2191.1741 | 13% | >98% |

^a^ measured by MALDI-TOF-MS; ^b^ measured by UV-Vis HPLC (λ = 254 nm).

**
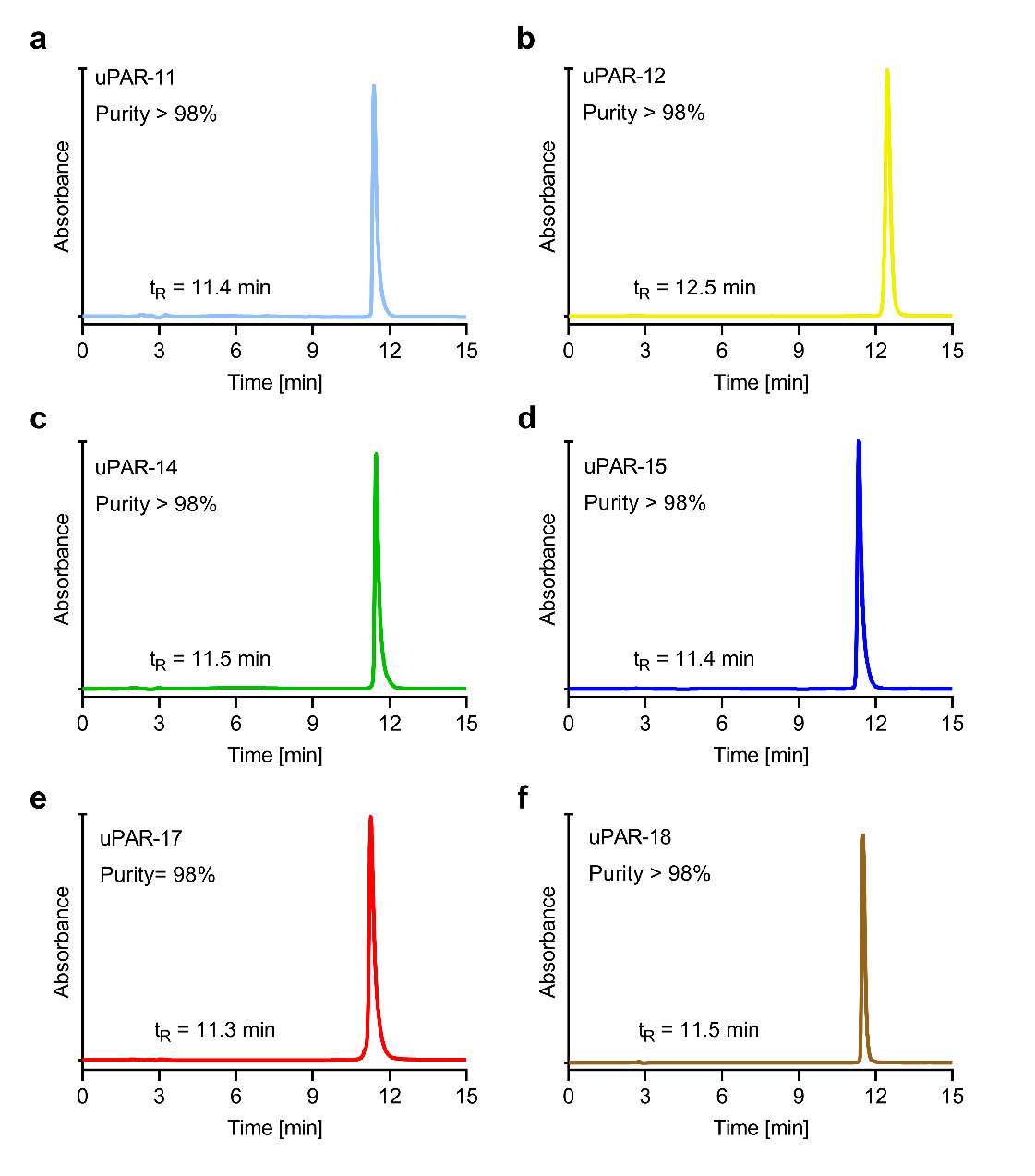
**

**Fig. S2** **a-f** Representative HPLC chromatograms of the uPAR-targeting peptides using a UV-Vis detector (λ = 254 nm). (**a**) uPAR-11, (**b**) uPAR-12, (**c**) uPAR-14, (**d**) uPAR-15, (**e**) uPAR-17 and (**f**) uPAR-18. Retention times (t_R_ = 11.3–12.5 min) and the chemical purities are indicated in the respective figures

**Fig. S3** HRMS of uPAR-11. Full-range spectrum (upper panel) with the red dot indicating the m/z value corresponding to the compound of interest. Zoomed-in comparison (lower panel) between the measured spectrum and the theoretical isotopic distribution

**Fig. S4** HRMS of uPAR-12. Full-range spectrum (upper panel) with the red dot indicating the m/z value corresponding to the compound of interest. Zoomed-in comparison (lower panel) between the measured spectrum and the theoretical isotopic distribution

**Fig. S5** HRMS of uPAR-14. Full-range spectrum (upper panel) with the red dot indicating the m/z value corresponding to the compound of interest. Zoomed-in comparison (lower panel) between the measured spectrum and the theoretical isotopic distribution

**Fig. S6** HRMS of uPAR-15. Full-range spectrum (upper panel) with the red dot indicating the m/z value corresponding to the compound of interest. Zoomed-in comparison (lower panel) between the measured spectrum and the theoretical isotopic distribution

**Fig. S7** HRMS of uPAR-17. Full-range spectrum (upper panel) with the red dot indicating the m/z value corresponding to the compound of interest. Zoomed-in comparison (lower panel) between the measured spectrum and the theoretical isotopic distribution

**Fig. S8** HRMS of uPAR-18. Full-range spectrum (upper panel) with the red dot indicating the m/z value corresponding to the compound of interest. Zoomed-in comparison (lower panel) between the measured spectrum and the theoretical isotopic distribution

**2. Radiolabeling procedure and stability of the radiopeptides in saline**

***Purpose:*** DOTA-AE105 and the new uPAR-targeting peptides were labeled with lutetium-177 and diluted in saline to assess their radiolytic stability.

***Methods:*** Stock solutions of the uPAR-targeting peptides were prepared in Milli-Q water at a concentration of 1 mM. Sodium acetate (0.5 M) was added to the respective stock solution of uPAR-14 (10% *v/v*), uPAR-15 (10% *v/v*) and uPAR-17 (11% *v/v*) to facilitate the dissolution of the peptides. The radiolabeling of the peptides was performed by the addition of lutetium-177 (no-carrier-added [^177^Lu]LuCl_3_ in 0.04 M HCl; ITM Medical Isotopes GmbH, Germany) to a 1:5 (*v*/*v*) mixture of sodium acetate (0.5 M) and HCl (0.05 M) at pH 4.5 followed by addition of the respective peptide stock solution (1 mM, i.e. 1 nmol corresp. 1 µL) to obtain molar activities up to 50 MBq/nmol. The reaction mixture was incubated for 10 min at 95 °C. Quality control of the radiolabeled peptides was performed using HPLC (Merck Hitachi HPLC system, Darmstadt, Germany, equipped with a radiodetector LB 508, Berthold Technologies) with a C18 reversed-phase column (Xterra^TM^ MS C-18, 5 µm, 15 cm x 4.6 cm, Waters, Milford, MA, USA). The radiopeptides were eluted by applying a linear gradient of Milli-Q water containing 0.1% TFA (95–20%) and ACN (5–80%) over 15 min at a flow rate of 1.0 mL/min.

The stability of the radiopeptides prepared at a molar activity of 50 MBq/nmol was investigated in saline at an activity concentration of 150 MBq/300 µL. A solution of L-ascorbic acid (3 mg in 20 µL) was added to the samples of the radiopeptides together with a sodium acetate solution (0.5 M, 30 µL) to obtain a pH value of ~5. Aliquots of these samples were investigated using HPLC after an incubation period of 1 h, 4 h and 24 h at room temperature. The integrated area of the peak representing the intact radiopeptide was expressed as the percentage of the sum of the integrated areas of all peaks present in the chromatogram. The determined amount of intact radiopeptide at the given timepoints was expressed as the percentage of intact radiopeptide relative to the initial value at t = 0, which was set as 100%.

***Results:*** The quality control revealed ≥95% radiochemical purity of the radiolabeled uPAR-targeting peptides prepared at a molar activity of 50 MBq/nmol (Fig. S9). The data of the stability assessment of the radiopeptides are reported in the main article and listed in Table S3.

**
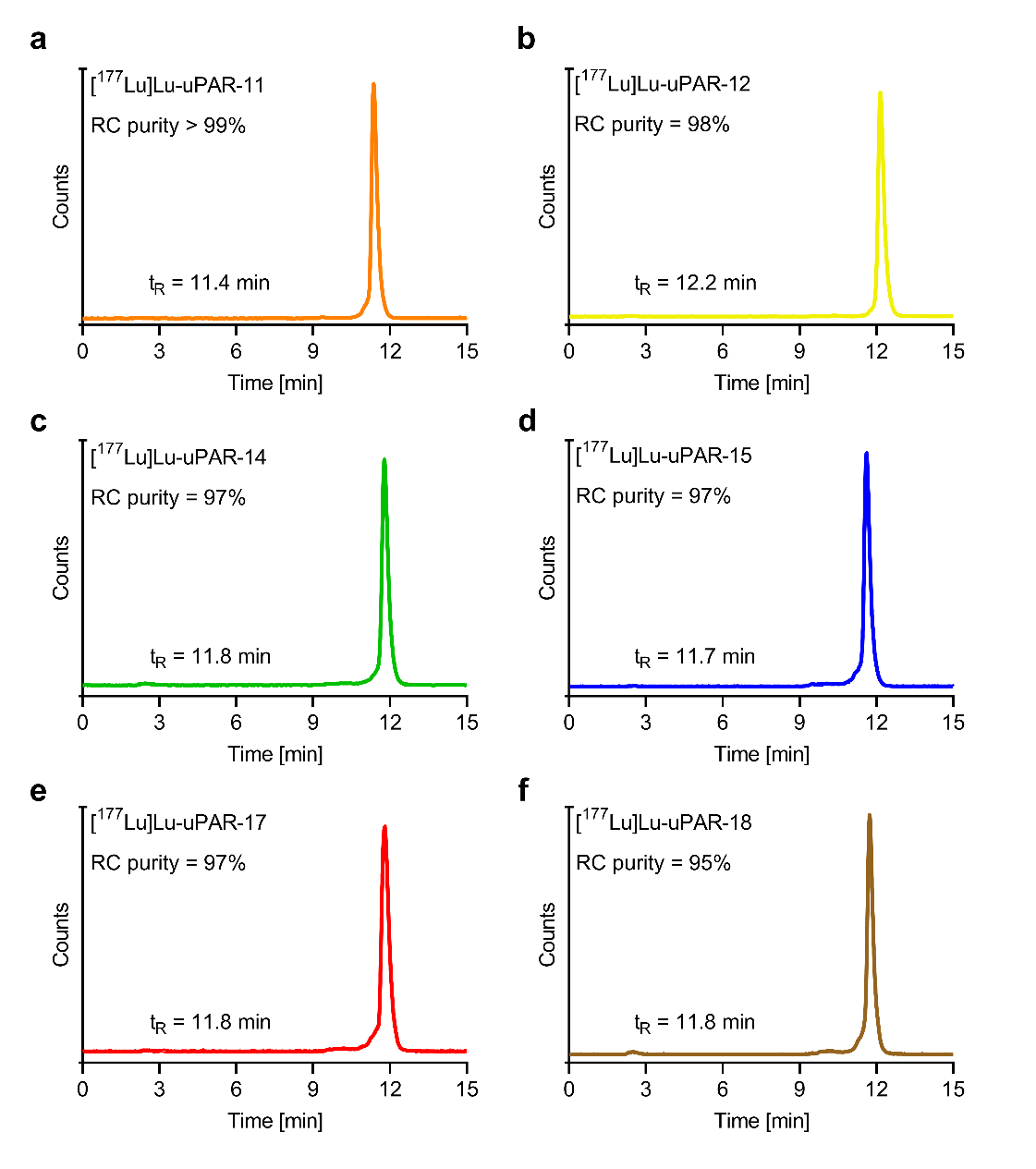
**

**Fig. S9** **a-f** Representative HPLC chromatograms of the uPAR-targeting peptides after radiolabeling with lutetium-177 (50 MBq/nmol). (**a**) [^177^Lu]Lu-uPAR-11, (**b**) [^177^Lu]Lu-uPAR-12, (**c**) [^177^Lu]Lu-uPAR-14, (**d**) [^177^Lu]Lu-uPAR-15, (**e**) [^177^Lu]Lu-uPAR-17 and (**f**) [^177^Lu]Lu-uPAR-18. Retention times (t_R_ = 11.4–12.2 min) and radiochemical (RC) purities are indicated on the graphs

**Table S3** Stability data of uPAR-targeting radiopeptides in the presence of l-ascorbic acid. The data are listed as the average ± SD of n = 3 experiments

| **Radiopeptide** | **Intact radiopeptide** | | |
| --- | --- | --- | --- |
|  | 1 h | 4 h | 24 h |
| [^177^Lu]Lu-DOTA-AE105^a^ | 98 ± 2 | 98 ± 1 | 96 ± 2 |
| [^177^Lu]Lu-uPAR-11 | 100 ± 1 | 100 ± 1 | 100 ± 1 |
| [^177^Lu]Lu-uPAR-12 | 100 ± 1 | 100 ± 1 | 96 ± 1 |
| [^177^Lu]Lu-uPAR-14 | 92 ± 4 | 91 ± 6 | 91 ± 5 |
| [^177^Lu]Lu-uPAR-15 | 97 ± 3 | 96 ± 2 | 91 ± 5 |
| [^177^Lu]Lu-uPAR-17 | 96 ± 1 | 94 ± 1 | 95 ± 3 |
| [^177^Lu]Lu-uPAR-18 | 98 ± 2 | 97 ± 5 | 96 ± 5 |

^a^Data reported with permission from Beyer *et al*. 2025, Mol Pharm 22:3242 [1]. Copyright 2025 American Chemical Society.

**3. Stability of the radiopeptides in mouse and human blood plasma**

***Purpose:*** The stability of the radiopeptides was assessed in mouse and human blood plasma as an indication of potential in vivo metabolism of the radiopeptides.

***Methods:*** A sample of each radiopeptide (50 MBq/nmol) was added to mouse blood plasma (Lot: 32321, Rockland Immunochemicals Inc., USA) or to human blood plasma (Stiftung Blutspende SRK Aargau-Solothurn, Switzerland) to obtain an activity concentration of 10 MBq/200 µL. The test samples were incubated at 37 °C for up to 24 h. Control samples referred to the radiopeptides which were instead diluted in saline at an activity concentration of 10 MBq/200 µL. Aliquots of each sample (2 µL, ~100 kBq) were taken after 1 h, 4 h and 24 h and investigated by means of thin layer chromatography (TLC). The employed method involved reversed-phase TLC plates (MERCK Analytical Chromatography, TLC Silica gel 60 RP-18 F_254_s) as stationary phase and ACN/citrate buffer (3:7; *v*/*v*; pH 5.5) as mobile phase. After the eluent reached the front line, the TLC plates were dried and exposed to a phosphor screen (Super resolution screen PSR10450013, PerkinElmer) for 1 min. The respective screen was developed using a storage phosphor system (Cyclone Plus, PerkinElmer) and analyzed using the OptiQuant Software (version 5.0, Bright Instrument Co Ltd., PerkinElmer).

***Results:*** The results are described in the main article and listed in Tables S4/S5.

**Table S4** Stability data of uPAR-targeting radiopeptides in mouse blood plasma. The data are listed as the average ± SD of n=3 experiments

| **Radiopeptide** | **Intact radiopeptide [%]** | | | | |
| --- | --- | --- | --- | --- | --- |
|  | 1 h | | 4 h | 24 h | |
| [^177^Lu]Lu-DOTA-AE105^a^ | 61 ± 6 | 13 ± 7 | | | 4.6 ± 2.2 |
| [^177^Lu]Lu-uPAR-11 | 99 ± 1 | 99 ± 1 | | | 95 ± 1 |
| [^177^Lu]Lu-uPAR-12 | 100 ± 1 | 99 ± 1 | | | 99 ± 1 |
| [^177^Lu]Lu-uPAR-14 | 100 ± 1 | 97 ± 4 | | | 99 ± 2 |
| [^177^Lu]Lu-uPAR-15 | 100 ± 1 | 99 ± 1 | | | 94 ± 2 |
| [^177^Lu]Lu-uPAR-17 | 100 ± 1 | 97 ± 2 | | | 61 ± 9 |
| [^177^Lu]Lu-uPAR-18 | 100 ± 1 | 99 ± 2 | | | 89 ± 3 |

^a^Data reported with permission from Beyer *et al*. 2025, Mol Pharm 22:3242 [1]. Copyright 2025 American Chemical Society.

**Table S5** Stability data of uPAR-targeting radiopeptides in human blood plasma. The data are listed as the average ± SD of n=3 experiments

| **Radiopeptide** | **Intact radiopeptide [%]** | | | | |
| --- | --- | --- | --- | --- | --- |
|  | 1 h | | 4 h | 24 h | |
| [^177^Lu]Lu-DOTA-AE105^a^ | 71 ± 3 | 13 ± 6 | | | 4.5 ± 1.2 |
| [^177^Lu]Lu-uPAR-11 | 99 ± 1 | 99 ± 1 | | | 95 ± 1 |
| [^177^Lu]Lu-uPAR-12 | 100 ± 1 | 99 ± 1 | | | 98 ± 1 |
| [^177^Lu]Lu-uPAR-14 | 100 ± 1 | 100 ± 1 | | | 97 ± 2 |
| [^177^Lu]Lu-uPAR-15 | 100 ± 1 | 99 ± 1 | | | 96 ± 1 |
| [^177^Lu]Lu-uPAR-17 | 100 ± 1 | 99 ± 1 | | | 96 ± 1 |
| [^177^Lu]Lu-uPAR-18 | 100 ± 1 | 99 ± 1 | | | 95 ± 2 |

^a^Data reported with permission from Beyer *et al*. 2025, Mol Pharm 22:3242 [1]. Copyright 2025 American Chemical Society.

**4. Determination of the *n*-octanol/PBS distribution coefficients (logD values)**

***Purpose:*** The *n*-octanol/PBS distribution coefficients (logD values) were determined as a measure for the hydrophilic/lipophilic character of the respective radiopeptides.

***Methods:*** The radiopeptides (50 MBq/nmol) were diluted in phosphate buffered saline (PBS) pH 7.4 to obtain an activity concentration of 10 MBq/500 µL. A sample of this radiopeptide solution (~0.5 MBq, 25 µL, 0.01 nmol) was added to a mixture of PBS pH 7.4 (1475 µL) and *n*-octanol (1500 µL). The vials were vortexed vigorously for 1 min followed by centrifugation (560 rcf, 25°C, 6 min) for phase separation. Aliquots were taken from each phase and measured in a γ-counter (Wallac Wizard 1480, PerkinElmer). The distribution coefficients were calculated as the logarithmic value of the ratio of counts per minute (cpm) measured in the *n*-octanol phase relative to the cpm measured in the PBS phase. The results were listed as average ± SD of the data obtained from 3 independent experiments, each performed with five replicates.

***Results:*** The data are reported in the main article.

**5. Determination of the plasma protein-binding properties**

***Purpose:*** The radiopeptides were investigated for their in vitro albumin-binding properties.

***Methods:*** The relative albumin-binding affinities of the radiopeptides in mouse blood plasma (Lot: 32321, Rockland Immunochemicals Inc., USA and human blood plasma (Stiftung Blutspende SRK Aargau-Solothurn, Switzerland) were determined using an ultrafiltration assay as previously reported [1]. The amount of mouse serum albumin (MSA) and human serum albumin (HSA) in mouse and human blood plasma was defined as 550 μM and 800 μM, respectively, based on albumin measurements using a dry chemistry analyzer (DRI-CHEM 4000i, FUJIFILM, Japan). A fixed amount of radiopeptide (50 MBq/nmol, ~300 kBq, 15 μL, 0.006 nmol) was added to 150 μL of mouse or human blood plasma and various dilutions were prepared using PBS (pH 7.4). This resulted in [MSA]-to-[radiopeptide] or [HSA]-to-[radiopeptide] molar concentration ratios of 0.01‒12500 and 0.01‒20000, respectively. The samples were incubated at 37 °C for 30 min. Ice-cold PBS (150 µL, pH 7.4) was added before loading the blood plasma samples on Amicon centrifugal filters (cut-off of 10 kDa; Merck Millipore) followed by centrifugation (14000 rcf, 4 °C, 30 min) to allow the separation of the plasma protein-bound from the unbound (free) fractions of each sample. The inserts of the filter devices were inverted and centrifuged at 200 rcf for 3 min to recover the protein-bound radiopeptide. The activity of the protein-bound fraction was measured using a γ-counter (Wallac Wizard 1480, PerkinElmer). The activity in the filtrate and filter unit was counted in the γ-counter and the counts were combined, assuming that the fraction retained in the filter membrane was not bound to proteins. The protein-bound fraction was expressed as percentage of the whole activity (i.e. plasma protein-bound activity, activity measured in the filtrate and activity measured in the filter (set as 100%)). The data were analyzed using a semi-logarithmic plot assuming a maximum binding of 100%. The Hill equation was fitted to the data points, and the half-maximum binding (B_50_) was determined based on the obtained binding curves using GraphPad Prism software (version 10.1.1). To quantitatively express and compare the albumin-binding properties of the radiopeptides, the relative albumin-binding affinity was expressed as the inverse half-maximal-binding (B_50_) of the respective radioligand to that of [^177^Lu]Lu-uPAR-11, which was set as 1.0. The results were presented as the value obtained from the average curve from 3 independent experiments.

***Results:*** The results are reported in the main article and listed in Table S6.

**Table S6** Inverse relative albumin-binding affinities of the radiopeptides in mouse and human blood plasma, normalized to the affinity of [^177^Lu]Lu-uPAR-11

| **Radiopeptide** | **Mouse blood plasma** | **Human blood plasma** |
| --- | --- | --- |
| [^177^Lu]Lu-DOTA-AE105 | 0.06^a^ | 0.02^a^ |
| [^177^Lu]Lu-uPAR-02 | 3.23^a^ | 4.61^a^ |
| [^177^Lu]Lu-uPAR-11 | 1.00^b^ | 1.00^b^ |
| [^177^Lu]Lu-uPAR-12 | 1.99 | 2.73 |
| [^177^Lu]Lu-uPAR-14 | 0.66 | 0.50 |
| [^177^Lu]Lu-uPAR-15 | 0.77 | 0.77 |
| [^177^Lu]Lu-uPAR-17 | 0.77 | 0.65 |
| [^177^Lu]Lu-uPAR-18 | 0.66 | 1.05 |

^a^Data reported with permission from Beyer *et al*. 2025, Mol Pharm 22:3242 [1]. Copyright 2025 American Chemical Society; ^b^The albumin-binding affinity was arbitrarily set as 1.00.

**6. Determination of uPAR-binding affinity (K_D_ values)**

***Purpose:*** The uPAR-binding affinity of the radiopeptides was determined experimentally for comparison of the radiopeptides.

***Methods:*** HEK-uPAR cells were seeded in poly-D-lysine-coated 48-well plates (0.25 × 10^6^ in 0.5 mL per well) using DMEM culture medium with supplements. The cells were incubated at 37 °C and 5% CO_2_ overnight to allow adhesion and growth. The well plates with HEK-uPAR cells were placed on ice for the entire experiment to prevent internalization of the radiopeptides. After removal of the medium, the HEK-uPAR cells were rinsed with cold PBS (0.5 mL, pH 7.4) followed by the addition of DMEM culture medium without supplements (450 µL/well) in the presence or absence of AE105 (final concentration 40 µM) as a receptor blocking agent. The cells were incubated for 30 min on ice. The respective radiopeptides (5 MBq/nmol) were diluted in PBS at peptide concentrations 10‒16000 nM. A volume of 50 µL from each dilution was added to each well to obtain peptide concentrations in the range of 1.01‒600 nM. The assay was performed at 4 °C on ice, to prevent internalization of the radiopeptides. The radiopeptides were incubated on cells for 1 h on a shaker before rinsing twice with PBS (0.5 mL, pH 7.4). Increasing ligand concentrations were used to approximate saturation of the binding sites (B_max_ value) at equilibrium. Ligand depletion effects of the ligand concentration in the supernatant of the cell samples were not taken into account. The cells were lysed using an aqueous NaOH solution (1 M, 600 µL) and the lysates were transferred to radioimmunoassay tubes for counting the activity in a γ-counter (Wallac Wizard 1480, PerkinElmer). The K_D_ values were determined by plotting the specific binding (total binding minus unspecific binding) against the molar concentration of the added radiopeptide using GraphPad Prism software (version 10.1.1). The results were then expressed as average ± SD of n = 3 independent experiments.

***Results:*** The K_D_ values are reported in the main article and representative saturation curves are shown in Fig. S10.


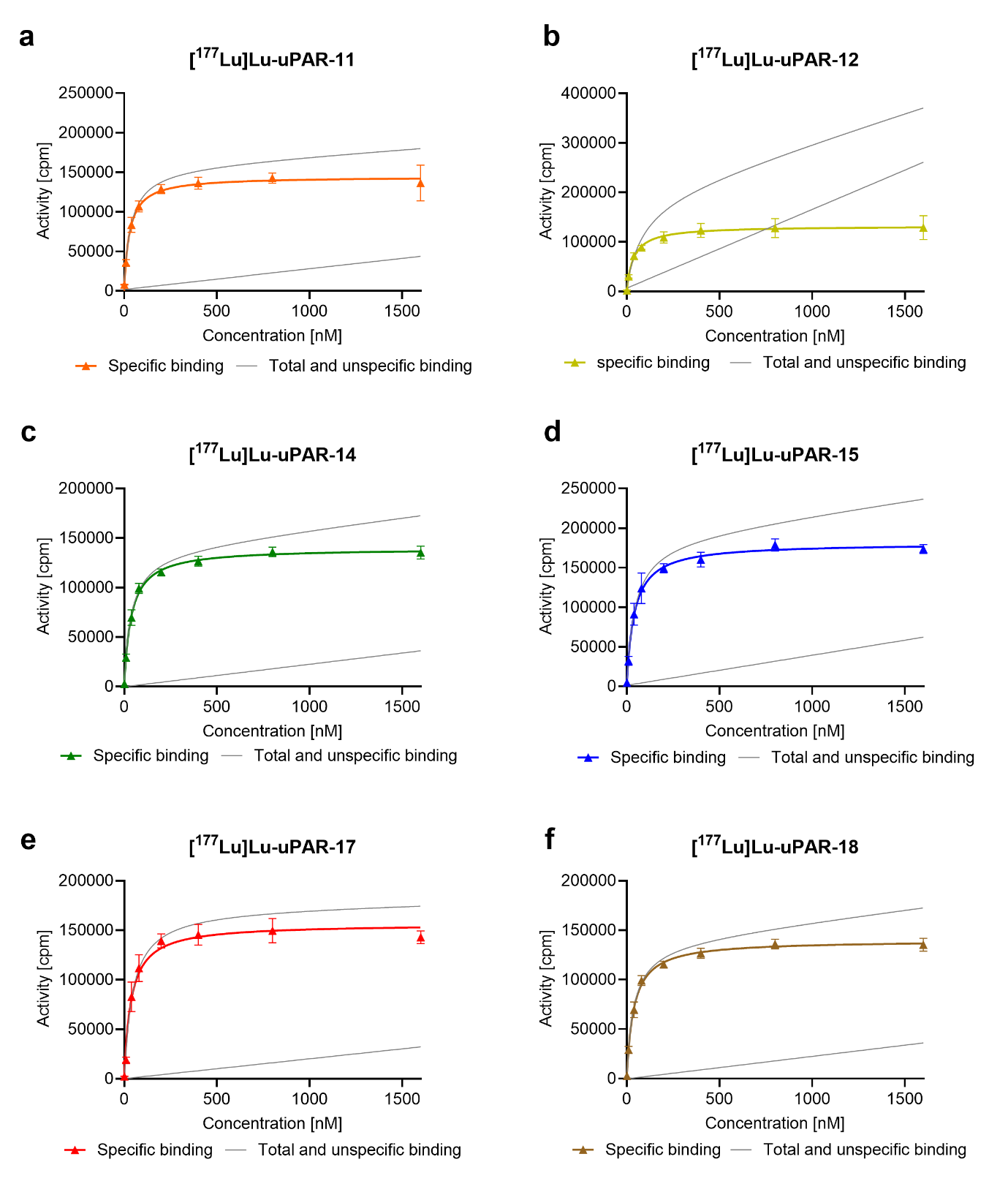


**Fig. S10 a‒f** Representative saturation curves of the radiopeptides using HEK-uPAR cells to determine their uPAR-binding affinity (K_D_ value). (**a**) [^177^Lu]Lu-uPAR-11, (**b**) [^177^Lu]Lu-uPAR-12, (**c**) [^177^Lu]Lu-uPAR-14, (**d**) [^177^Lu]Lu-uPAR-15, (**e**) [^177^Lu]Lu-uPAR-17 and (**f**) [^177^Lu]Lu-uPAR-18

**7. SPECT/CT imaging studies and quantification**

***Purpose:*** Single photon emission computed tomography/computed tomography (SPECT/CT) imaging studies were performed to determine the whole-body tissue distribution profile of the radiopeptides in mice over a 24 h period.

***Methods:*** SPECT/CT experiments were performed with CD1 nude mice (Crl:CD1-Foxn1^nu^) approximately 3‒9 weeks after HEK-uPAR cell inoculation. The mice were scanned 1 h, 4 h and 24 h post injection (p.i.) of the respective radiopeptide (25 MBq, 0.5 nmol, 100 µL, diluted in 0.9% NaCl containing 0.05% bovine serum albumin (BSA)). Imaging studies were performed using a four-head, multiplexing, multi-pinhole small-animal SPECT/CT camera (NanoSPECT/CT^TM^, Mediso Medical Imaging Systems, Budapest, Hungary). Each head was outfitted with a tungsten-based aperture of nine 1.4 mm-diameter pinholes and a thickness of 10 mm. CT scans of 7‒9 min duration were followed by SPECT scans of 45‒50 min. The images were acquired using Nucline Software (version 10.2, Mediso Ltd., Budapest, Hungary). The real-time CT reconstruction used a cone-beam filtered backprojection. The reconstruction of SPECT data was performed with HiSPECT software (version 1.4.3049, Scivis GmbH, Göttingen, Germany) using γ-energies of 56.1 keV (± 10%), 112.9 keV (± 10%) and 208.4 keV (± 10%) for lutetium-177. The images were prepared using VivoQuant post-processing software (version 3.5, inviCRO Imaging Services and Software, Boston, USA). A Gauss post-reconstruction filter (full width at half maximum, 1.0 mm) was applied and the scale of activity was set as indicated on the images (minimum value = 0.2 Bq/voxel, maximum value = 20 Bq/voxel).

***Results:*** The results are reported in the main article.

**8. Biodistribution studies**

***Purpose:*** Biodistribution studies were performed with HEK-uPAR xenograft-bearing mice to quantitatively assess the accumulation of activity in the xenografts as well as in selected organs and tissues.

***Methods:*** The methods are reported in the main article.

***Results:*** The results are reported in the main article and the data are listed in Tables S7-S14.

**Table S7** Biodistribution data and xenograft-to-background ratios of accumulated activity obtained in HEK-uPAR xenograft-bearing mice at 1 h, 4 h and 24 h after injection of [^177^Lu]Lu-DOTA-AE105. Decay-corrected data of accumulated activity are shown as % IA/g tissue, representing the average ± SD of n=3 mice

|  | **[^177^Lu]Lu-DOTA-AE105** | | |  |
| --- | --- | --- | --- | --- |
|  | **1 h p.i.** | **4 h p.i.^a^** | **24 h p.i.^a^** | |
|  | [%IA/g] | [%IA/g] | [%IA/g] | |
| Blood | 0.92 ± 0.40 | 0.08 ± 0.01 | <0.03 | |
| Heart | 0.38 ± 0.16 | 0.06 ± 0.01 | 0.04 ± 0.01 | |
| Lung | 1.2 ± 0.40 | 0.30 ± 0.02 | 0.21 ± 0.01 | |
| Spleen | 0.47 ± 0.17 | 0.18 ± 0.02 | 0.23 ± 0.04 | |
| Kidneys | 4.2 ± 0.8 | 1.5 ± 0.1 | 1.3 ± 0.1 | |
| Stomach | 0.39 ± 0.20 | 0.15 ± 0.03 | 0.17 ± 0.03 | |
| Pancreas | 0.26 ± 0.08 | 0.06 ± 0.01 | 0.06 ± 0.01 | |
| Intestines | 0.52 ± 0.10 | 0.31 ± 0.03 | 0.18 ± 0.02 | |
| Liver | 0.40 ± 0.14 | 0.10 ± 0.01 | 0.17 ± 0.01 | |
| Muscle | 0.14 ± 0.06 | <0.03 | <0.03 | |
| Bone | 0.35 ± 0.11 | 0.38 ± 0.03 | 0.84 ± 0.10 | |
| HEK-uPAR xenograft | 1.9 ± 0.7 | 0.87 ± 0.05 | 0.40 ± 0.19 | |
| Salivary glands | 0.35 ± 0.13 | 0.09 ± 0.01 | 0.09 ± 0.01 | |
| Brain | <0.03 | <0.03 | <0.03 | |
|  | Xenograft-to-background ratios | | | |
| Xgrft-to-kidney | 0.46 ± 0.07 | 0.58 ± 0.03 | 0.33 ± 0.14 | |
| Xgrft-to-blood | 2.2 ± 0.2 | 11 ± 1 | 32 ± 13 | |
| Xgrft-to-liver | 4.9 ± 0.1 | 8.6 ± 0.4 | 2.4 ± 0.9 | |

^a^Data reported with permission from Beyer *et al*. 2025, Mol Pharm 22:3242 [1]. Copyright 2025 American Chemical Society.

**Table S8** Biodistribution data and xenograft-to-background ratios obtained in HEK-uPAR xenograft-bearing mice at 1 h, 4 h, 24 h and 48 h after injection of [^177^Lu]Lu-uPAR-11. Decay-corrected data of accumulated activity are shown as % IA/g tissue, representing the average ± SD (n = 3)

|  | **[^177^Lu]Lu-uPAR-11** | | | |
| --- | --- | --- | --- | --- |
|  | **1 h p.i.** | **4 h p.i.** | **24 h p.i.** | **48 h p.i.** |
|  | [%IA/g] | [%IA/g] | [%IA/g] | [%IA/g] |
| Blood | 13 ± 2 | 5.0 ± 0.6 | <0.03 | <0.03 |
| Heart | 4.9 ± 0.3 | 1.8 ± 0.2 | 0.12 ± 0.02 | 0.08 ± 0.01 |
| Lung | 7.0 ± 1.3 | 3.1 ± 0.2 | 0.29 ± 0.08 | 0.17 ± 0.02 |
| Spleen | 2.2 ± 0.3 | 1.1 ± 0.1 | 0.49 ± 0.05 | 0.37 ± 0.05 |
| Kidneys | 6.8 ± 0.9 | 4.0 ± 0.4 | 1.7 ± 0.1 | 1.1 ± 0.1 |
| Stomach | 1.8 ± 0.5 | 0.75 ± 0.19 | 0.12 ± 0.01 | 0.09 ± 0.00 |
| Pancreas | 1.7 ± 0.2 | 0.69 ± 0.05 | 0.09 ± 0.02 | 0.07 ± 0.02 |
| Intestines | 1.8 ± 0.2 | 0.93 ± 0.11 | 0.19 ± 0.01 | 0.14 ± 0.03 |
| Liver | 4.5 ± 0.3 | 1.8 ± 0.2 | 0.33 ± 0.03 | 0.25 ± 0.02 |
| Muscle | 1.4 ± 0.3 | 0.63 ± 0.09 | 0.05 ± 0.00 | 0.04 ± 0.01 |
| Bone | 1.7 ± 0.4 | 0.73 ± 0.06 | 0.19 ± 0.03 | 0.11 ± 0.02 |
| HEK-uPAR xenograft | 13 ± 2 | 16 ± 2 | 7.9 ± 0.9 | 5.3 ± 0.7 |
| Salivary glands | 2.9 ± 0.5 | 1.3 ± 0.2 | 0.17 ± 0.02 | 0.18 ± 0.04 |
| Brain | 0.28 ± 0.03 | 0.12 ± 0.23 | <0.03 | <0.03 |
|  | Xenograft-to-background ratios | | | |
| Xgrft-to-kidney | 2.0 ± 0.2 | 3.9 ± 0.2 | 4.7 ± 0.2 | 4.9 ± 0.7 |
| Xgrft-to-blood | 1.0 ± 0.1 | 3.1 ± 0.1 | 274 ± 25 | 311 ± 18 |
| Xgrft-to-liver | 3.0 ± 0.3 | 8.7 ± 0.1 | 24 ± 2 | 22 ± 2 |

**Table S9** Biodistribution data and xenograft-to-background ratios obtained in HEK-uPAR xnograft-bearing mice at 4 h and 24 h after injection of [^177^Lu]Lu-uPAR-12. Decay-corrected data of accumulated activity are shown as % IA/g tissue, representing the average ± SD (n = 3)

|  | **[^177^Lu]Lu-uPAR-12** | |
| --- | --- | --- |
|  | **4 h p.i.** | **24 h p.i.** |
|  | [%IA/g] | [%IA/g] |
| Blood | 12 ± 1 | 3.4 ± 1.0 |
| Heart | 4.0 ± 0.3 | 1.6 ± 0.2 |
| Lung | 7.2 ± 1.3 | 2.6 ± 0.6 |
| Spleen | 1.9 ± 0.1 | 2.0 ± 0.6 |
| Kidneys | 3.9 ± 0.1 | 4.3 ± 0.3 |
| Stomach | 1.4 ± 0.1 | 0.52 ± 0.16 |
| Pancreas | 1.4 ± 0.2 | 0.79 ± 0.24 |
| Intestines | 1.5 ±0.1 | 0.76 ± 0.12 |
| Liver | 5.3 ± 0.3 | 4.2 ± 0.3 |
| Muscle | 1.2 ± 0.1 | 0.60 ± 0.22 |
| Bone | 1.4 ± 0.1 | 0.78 ± 0.15 |
| HEK-uPAR xenograft | 6.0 ± 0.2 | 5.7 ± 0.7 |
| Salivary glands | 2.7 ± 0.2 | 1.40 ± 0.2 |
| Brain | 0.24 ± 0.01 | 0.10 ± 0.03 |
|  | Xenograft-to-background ratios | |
| Xgrft-to-kidney | 1.6 ± 0.1 | 1.3 ± 0.1 |
| Xgrft-to-blood | 0.49 ± 0.03 | 1.7 ± 0.2 |
| Xgrft-to-liver | 1.1 ± 0.1 | 1.3 ± 0.1 |

**Table S10** Biodistribution data and xenograft-to-background ratios obtained in HEK-uPAR xenograft-bearing mice at 4 h and 24 h after injection of [^177^Lu]Lu-uPAR-14. Decay-corrected data of accumulated activity are shown as % IA/g tissue, representing the average ± SD (n = 3)

|  | **[^177^Lu]Lu-uPAR-14** | |
| --- | --- | --- |
|  | **4 h p.i.** | **24 h p.i.** |
|  | [%IA/g] | [%IA/g] |
| Blood | 4.5 ± 0.2 | 0.06 ± 0.01 |
| Heart | 1.7 ± 0.1 | 0.15 ± 0.01 |
| Lung | 3.0 ± 0.6 | 0.30 ± 0.02 |
| Spleen | 0.99 ± 0.08 | 0.54 ± 0.11 |
| Kidneys | 4.0 ± 0.3 | 2.0 ± 0.3 |
| Stomach | 0.69 ± 0.13 | 0.16 ± 0.03 |
| Pancreas | 0.68 ± 0.03 | 0.12 ± 0.01 |
| Intestines | 0.81 ± 0.04 | 0.21 ± 0.03 |
| Liver | 2.0 ± 0.2 | 0.47 ± 0.05 |
| Muscle | 0.51 ± 0.02 | 0.05 ± 0.01 |
| Bone | 0.66 ± 0.09 | 0.19 ± 0.03 |
| HEK-uPAR xenograft | 9.0 ± 0.5 | 3.4 ± 0.3 |
| Salivary glands | 1.1 ± 0.1 | 0.26 ± 0.03 |
| Brain | 0.10 ± 0.01 | <0.03 |
|  | Xenograft-to-background ratios | |
| Xgrft-to-kidney | 2.3 ± 0.2 | 1.8 ± 0.3 |
| Xgrft-to-blood | 2.0 ± 0.2 | 61 ± 12 |
| Xgrft-to-liver | 4.5 ± 0.3 | 7.4 ± 1.0 |

**Table S11** Biodistribution data and xenograft-to-background ratios obtained in HEK-uPAR xenograft-bearing mice at 4 h and 24 h after injection of [^177^Lu]Lu-uPAR-15. Decay-corrected data of accumulated activity are shown as % IA/g tissue, representing the average ± SD (n = 3)

|  | **[^177^Lu]Lu-uPAR-15** | |
| --- | --- | --- |
|  | **4 h p.i.** | **24 h p.i.** |
|  | [%IA/g] | [%IA/g] |
| Blood | 4.5 ± 0.5 | 0.04 ± 0.01 |
| Heart | 1.6 ± 0.2 | 0.13 ± 0.01 |
| Lung | 2.6 ± 0.4 | 0.27 ± 0.06 |
| Spleen | 0.93 ± 0.09 | 0.51 ± 0.23 |
| Kidneys | 3.9 ± 0.6 | 2.0 ± 0.3 |
| Stomach | 0.67 ± 0.11 | 0.12 ± 0.03 |
| Pancreas | 0.61 ± 0.10 | 0.09 ± 0.02 |
| Intestines | 0.74 ± 0.15 | 0.16 ± 0.03 |
| Liver | 1.6 ± 0.2 | 0.50 ± 0.11 |
| Muscle | 0.41 ± 0.10 | 0.05 ± 0.01 |
| Bone | 0.66 ± 0.05 | 0.12 ± 0.03 |
| HEK-uPAR xenograft | 12 ± 1 | 3.1 ± 0.8 |
| Salivary glands | 1.2 ± 0.2 | 0.21 ± 0.03 |
| Brain | 0.10 ± 0.02 | <0.03 |
|  | Xenograft-to-background ratios | |
| Xgrft-to-xidney | 3.1 ± 0.4 | 1.7 ± 0.5 |
| Xgrft-to-blood | 2.7 ± 0.2 | 79 ± 17 |
| Xgrft-to-liver | 7.7 ± 0.7 | 6.5 ± 1.7 |

**Table S12** Biodistribution data and xenograft-to-background ratios obtained in HEK-uPAR xenograft-bearing mice at 4 h and 24 h after injection of [^177^Lu]Lu-uPAR-17. Decay-corrected data of accumulated activity are shown as % IA/g tissue, representing the average ± SD (n = 3)

|  | **[^177^Lu]Lu-uPAR-17** | |
| --- | --- | --- |
|  | **4 h p.i.** | **24 h p.i.** |
|  | [%IA/g] | [%IA/g] |
| Blood | 6.0 ± 1.3 | <0.03 |
| Heart | 2.2 ± 0.4 | 0.07 ± 0.01 |
| Lung | 3.0 ± 0.7 | 0.12 ± 0.02 |
| Spleen | 1.0 ± 0.2 | 0.13 ± 0.02 |
| Kidneys | 4.6 ± 0.3 | 1.3 ± 0.2 |
| Stomach | 0.73 ± 0.29 | 0.05 ± 0.01 |
| Pancreas | 0.82 ± 0.17 | 0.04 ± 0.01 |
| Intestines | 0.87 ± 0.18 | 0.05 ± 0.01 |
| Liver | 1.7 ± 0.2 | 0.21 ± 0.03 |
| Muscle | 0.57 ± 0.10 | <0.03 |
| Bone | 0.82 ± 0.10 | 0.06 ± 0.01 |
| HEK-uPAR xenograft | 8.0 ± 0.7 | 2.1 ± 0.5 |
| Salivary glands | 1.2 ± 0.3 | 0.12 ± 0.03 |
| Brain | 0.13 ± 0.04 | <0.03 |
|  | Xenograft-to-background ratios | |
| Xgrft-to-kidney | 1.7 ± 0.1 | 1.7 ± 0.2 |
| Xgrft-to-blood | 1.4 ± 0.2 | 62 ± 5 |
| Xgrft-to-liver | 4.7 ± 0.2 | 10 ± 3 |

**Table S13** Biodistribution data and xenograft-to-background ratios obtained in HEK-uPAR xenograft-bearing mice at 4 h and 24 h after injection of [^177^Lu]Lu-uPAR-18. Decay-corrected data of accumulated activity are shown as % IA/g tissue, representing the average ± SD (n = 3)

|  | **[^177^Lu]Lu-uPAR-18** | |
| --- | --- | --- |
|  | **4 h p.i.** | **24 h p.i.** |
|  | [%IA/g] | [%IA/g] |
| Blood | 3.4 ± 0.7 | 0.05 ± 0.00 |
| Heart | 1.3 ± 0.2 | 0.12 ± 0.01 |
| Lung | 2.1 ± 0.4 | 0.29 ± 0.02 |
| Spleen | 0.77 ± 0.01 | 0.42 ± 0.04 |
| Kidneys | 3.7 ± 0.4 | 2.1 ± 0.2 |
| Stomach | 0.54 ± 0.12 | 0.11 ± 0.02 |
| Pancreas | 0.63 ± 0.27 | 0.09 ± 0.01 |
| Intestines | 0.58 ± 0.14 | 0.17 ± 0.02 |
| Liver | 1.2 ± 0.1 | 0.38 ± 0.06 |
| Muscle | 0.37 ± 0.03 | 0.06 ± 0.01 |
| Bone | 0.50 ± 0.04 | 0.19 ± 0.01 |
| HEK-uPAR xenograft | 14 ± 1 | 7.3 ± 0.4 |
| Salivary glands | 0.87 ± 0.20 | 0.23 ± 0.03 |
| Brain | 0.08 ± 0.01 | <0.03 |
|  | Xenograft-to-background ratios | |
| Xgrft-to-kidney | 3.8 ± 0.3 | 3.5 ± 0.4 |
| Xgrft-to-blood | 4.1 ± 0.7 | 144 ± 4 |
| Xgrft-to-liver | 11 ± 1 | 20 ± 3 |

**Table S14** Area under the curve (AUC) values (± standard error) and ratios based on non-decay-corrected biodistribution data of the uPAR radiopeptides from n=3 mice per timepoint

|  | AUC_0h→48h_ values [% IA/g*h] | |
| --- | --- | --- |
|  | **[^177^Lu]Lu-DOTA-AE105** | **[^177^Lu]Lu-uPAR-11** |
| HEK-uPAR xenograft | 26 ± 3 | 410 ± 21 |
| Blood | 28 ± 1 | 109 ± 6 |
| Kidneys | 62 ± 2 | 103 ± 5 |
| Liver | 6.9 ± 0.2 | 38 ± 2 |

**9.** **In vivo stability studies**

***Purpose:*** The in vivo stability of [^177^Lu]Lu-uPAR-11 was investigated in immunocompetent FVB mice by analyzing urine, blood plasma, liver and kidneys at 1 h and 4 h after radiopeptide injection for comparison with the data previously obtained for [^177^Lu]Lu-DOTA-AE105 [1].

***Methods:*** FVB mice (FVB/NCrl) (n=2) were injected with [^177^Lu]Lu-uPAR-11 (25 MBq, 0.5 nmol, in 100 µL 0.05% BSA in saline). Urine was collected at 1 h and 4 h p.i. before sacrificing the mice. Subsequently, blood was sampled from the heart and liver and kidneys were collected. The blood was centrifuged (200 rcf, 4 °C, 10 min) to obtain blood plasma. A drop of urine and blood plasma were analyzed using the TLC method employed for in vitro blood plasma stability as described above. Both kidneys and a part of the liver (~200 mg) were cut into pieces, homogenized using 200‒300 μL buffer solution (ice-cold methanol with 0.025% NH_4_OH) and centrifuged (2300 rcf, 4° C, 5 min) as described by Beyer *et al*. [2]. The supernatant of each sample was developed using the TLC system described above. The injection solution was spotted as a control on each TLC plate. The TLC plates were exposed to a phosphor screen (Super resolution screen PSR10450013, PerkinElmer) followed by development using a storage phosphor system (Cyclone Plus, PerkinElmer). The quantification of the signals was carried out using the OptiQuant software (version 5.0, Bright Instrument Co Ltd., PerkinElmer). The obtained chromatograms were analyzed by determination of the peak area of the radiolabeled peptide as well as degradation products of unknown structure. The quantity of the intact [^177^Lu]Lu-uPAR-11 was expressed as percentage of the sum of integrated peak areas of the entire chromatogram.

***Results:*** The results are reported in the main article and in Table S15.

**Table S15** Metabolic stability of radiopeptides after injection into FVB mice. Data is shown as average from samples of n = 2 mice per radiopeptide and timepoint

|  |  | **[^177^Lu]Lu-DOTA-AE105^a^** | | **[^177^Lu]Lu-uPAR-11** | |
| --- | --- | --- | --- | --- | --- |
|  |  | 1 h p.i. | 4 h p.i. | 1 h p.i. | 4 h p.i. |
| Blood | Intact radiopeptide | <1.0 % | <1.0 % | 66 | 34 |
|  | Radiometabolite | >99% | >99% | 34 | 66 |
| Liver | Intact radiopeptide | <1.0 % | <1.0 % | 32 | 17 |
|  | Radiometabolite | >99% | >99% | 68 | 83 |
| Kidneys | Intact radiopeptide | <1.0 % | <1.0 % | 19 | 11 |
|  | Radiometabolite | >99% | >99% | 81 | 89 |
| Urine | Intact radiopeptide | 1.4 % | 2.3% | 1.9 | 4.5 |
|  | Radiometabolite | 99% | 98% | 98 | 95 |

^a^Data reported with permission from Beyer *et al*. 2025, Mol Pharm 22:3242 [1]. Copyright 2025 American Chemical Society.

**10. Computational studies**

***Purpose:*** The compatibility of the modifications realized in the new radiopeptide series with the uPAR-binding was confirmed by visual inspection of a uPAR model with bound Lu-uPAR-11.

***Methods:*** The molecular model of Lu-uPAR-11 bound to the human uPAR was built using a template of the model previously prepared for Lu-uPAR-02 [1]. A molecular graphics software (ChemDoodle 3D (version 7.5.1), iChemLabs, LLC) was used to replace the *p*-iodophenyl group of Lu-uPAR-02 with a *p*-tolyl group and generate Lu-uPAR-11, and its geometry was then optimized using the MMFF94 force field.

***Results:*** The generated uPAR model with bound Lu-uPAR-11 (Fig. S11) confirmed that all structural variations in the new series of derivatives involved linker region, the albumin binder and a macrocyclic chelator, that do not take strong interactions with the uPAR-binding protein. Therefore, these modifications were not expected to influence the uPAR-binding affinity of the new radiopeptide series substantially. This was experimentally confirmed by the determination of K_D_ values which were all in a similar range.

**
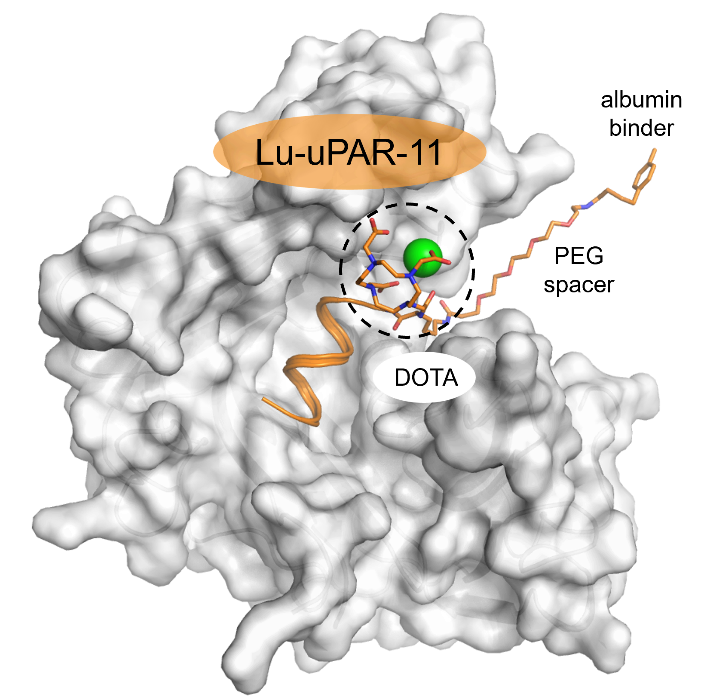
**

**Fig. S11** Molecular model of Lu-uPAR-11 bound to uPAR. The peptide conjugate is depicted in orange, lutetium as a green sphere, and the molecular surface of the protein in grey. The PEG spacer of Lu-uPAR-11 occupies the crevice between the *N*- and *C*-termini of the receptor, enabling the albumin binder to protrude from the protein surface

**References**

1. Beyer D, Vaccarin C, Schmid JV, Deberle LM, Deupi X, Schibli R, et al. Design and preclinical evaluation of novel uPAR-targeting radiopeptides modified with an albumin-binding entity. Mol Pharm. 2025. doi:10.1021/acs.molpharmaceut.5c00135.

2. Beyer D, Vaccarin C, Deupi X, Mapanao AK, Cohrs S, Sozzi-Guo F, et al. A tool for nuclear imaging of the SARS-CoV-2 entry receptor: molecular model and preclinical development of ACE2-selective radiopeptides. EJNMMI Res. 2023;13:32. doi:10.1186/s13550-023-00979-2.
